# Role of Stereochemistry in Controlling Magnetic Behavior in Polymeric Materials

**DOI:** 10.1021/jacs.6c01683

**Published:** 2026-05-18

**Authors:** Naushad Ahmed, Akhil Kumar Singh, Mani Sengoden, Marcetta Y. Darensbourg, Donald J. Darensbourg

**Affiliations:** Department of Chemistry, 14736 Texas A&M University, College Station, Texas 77843, United States of America

## Abstract

Stable radical polymers are emerging as important functional materials for a variety of applications such as spintronics, electrical conductivity, and quantum information technologies. To better understand the magnetic behavior of such polymers, we have developed a facile synthesis of these polymeric materials via copolymerization of COS with the biobased epoxide vanillin glycidyl ether (VGE, R-VGE, or S-VGE). Importantly, this monomer contains an aldehyde-reactive group, which allows for the addition of 4-amino-TEMPO via Schiff base condensation before polymerization (**PMTC-1′, R-PMTC-1′,** and **S-PMTC-1′** polymers from VGE-TEMPO, R-VGE-TEMPO, or S-VGE-TEMPO, respectively) or postpolymerization (**PMTC-1, R-PMTC-1,** and **S-PMTC-1** polymers from VGE, R-VGE, or S-VGE, respectively). We demonstrated that the polymers’ tacticity controls the magnetic behaviors of these radical polymers under various applied magnetic fields. Interestingly, we noticed the magnetic switching in *isotactic* **R-PMTC-1** (and **R-PMTC-1′**) and **S-PMTC-1** (and **S-PMTC-1′**). These magnetic switchable polymers are expected to be suitable for various device applications.

## Graphical abstract

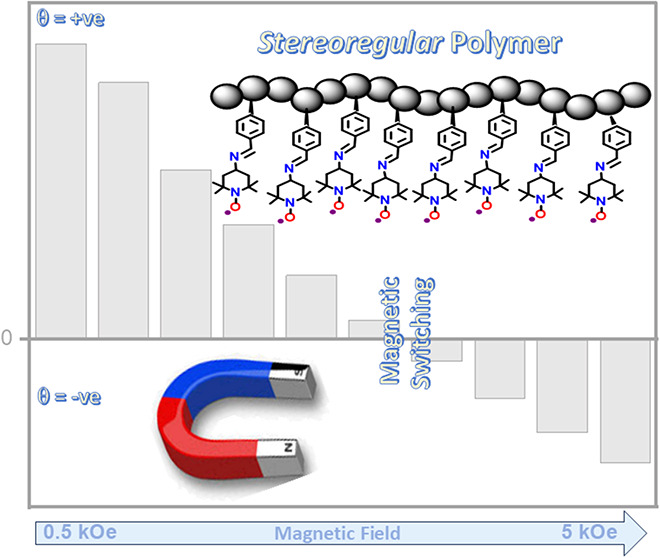

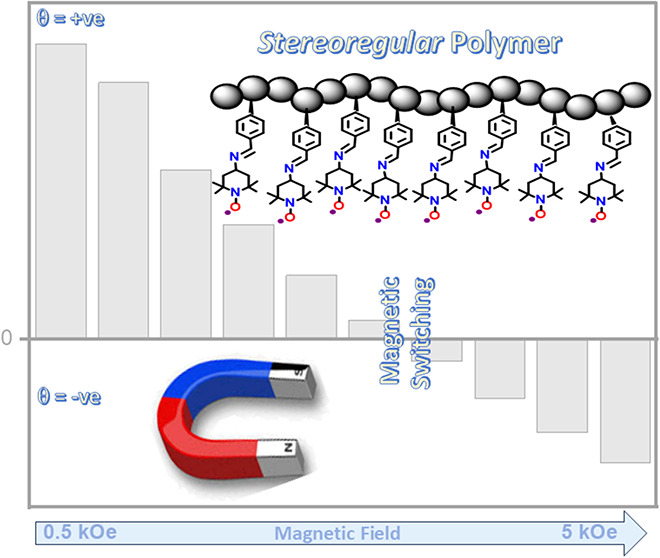

## 1 Introduction

Presently, conjugated and nonconjugated radical polymers bearing the air-stable open-shell radical pendant functionality in their side chains have garnered special interest in investigating the electrical conductivity,^1,2^ spintronics,^3,4^ photovoltaic,^5,6^ energy storage,^7–9^ , and magnetic behaviors^10,11^ for their wide range of applications. These properties can be tuned and modulated in these polymers by controlling (i) the radical–radical distance and (ii) the regio- and stereoselectivity. In a polymer, the closer radical–radical distance facilitates electrical conductivity by the fast-spin transfer via a hopping mechanism, as well as may also promote the stronger magnetic exchange.^12^ On the other hand, the regular arrangement of the radicals in stereoregular *isotactic* polymers further provides an easier electron transfer pathway compared to irregular *atactic* polymers, hence facilitating better conductivity.^3^ Although we have explored the electrical conductivity and tacticity of radical polymers,^13^ to the best of our knowledge, the correlation between tacticity and the nature of magnetic exchange has rarely or not yet been explored, especially using sustainable polycarbonate and/or poly­(monothiocarbonate) radical polymers. In this report, we preferentially adopted a postsynthetic installation of 4-amino-2,2,6,6-tetramethylpiperidine-1-oxyl (4-amino-TEMPO) stable free radicals via Schiff base condensation with the pendant aldehyde group of the *atactic* and *isotactic* sustainable poly­(monothiocarbonates) to produce radical polymers. These *atactic* and *isotactic* radical poly­(monothiocarbonates) can also be produced from the preinstalled TEMPO epoxy monomers. We have performed detailed experimental and computational studies to establish the correlation between the nature of magnetic interactions and the tacticity. The advantage of the postsynthetic installation of free radicals at the polymer’s side chain over polymers grown using a radical monomer is that it allows the polymers to be grown with the desired molecular weight and chain length as required. In contrast, in the latter case, the presence of free radicals in the monomer may interfere with and restrict the further growth of the polymer.

## 2 Experimental Section

All of the reagents (vanillin, epichlorohydrin, and 4-amino-TEMPO) and solvents (dichloromethane, methanol, and tetrahydrofuran) for the reaction were purchased from commercial sources such as AmBeed and Sigma-Aldrich. Solvents and reagents were used as received for the synthesis of the epoxy monomers. For polymer synthesis, inert conditions were maintained, and solvents were dried according to the reported procedure.^14^ Postpolymerization modification of pendant aldehyde was performed under aerobic conditions. For the determination of the molecular weights of polymers, we used gel permeation chromatography (GPC). We used a Quantum Design MPMS3 SQUID magnetometer for magnetic measurements, which were performed on the bulk samples tightly wrapped in Teflon tape, and a straw was used as a sample holder. We used a Bruker Elexsys E500 console spectrometer for recording electron paramagnetic resonance (EPR) spectra.

## 3 Results and Discussion

Pendant functional groups, such as vinyl (−CH=CH_2_) and formyl (−CHO), in a polymer’s side chains have garnered attention due to their postfunctionalization properties and suitability in 3D printing, biocompatible hydrogels, and other applications.^15,16^ Inspired by these important applications, herein, we report the utilization of the epoxide monomer 3-methoxy-4-(oxiran-2-ylmethoxy)­benzaldehyde (VGE, vanillin glycidyl ether) in coupling reactions with CO_2_ or COS. The epoxide monomer VGE was prepared from biobased chemicals, vanillin and epichlorohydrin, in good yield as described in Scheme 1, and its purity was ensured by column chromatography using silica gel and a hexane and ethyl acetate solvent mixture in a 2:1 ratio.^17^ The spectroscopic techniques, such as ^1^H NMR and mass spectrometry (Figures S1 and S2 in ESI) of the VGE monomer, confirm its purity. It should be noted that epichlorohydrin is synthesized commercially from glycerol, a byproduct of biodiesel production, known as the *Epicerol* process.^18,19^ The radical VGE-TEMPO monomer was prepared via Schiff base condensation reaction with 4-amino-TEMPO as described in Scheme 1. Following a similar synthetic procedure, the R-VGE and S-VGE enantiomers were prepared from the corresponding epichlorohydrin, and from them, R-VGE-TEMPO and S-VGE-TEMPO were derived, respectively.

**Scheme 1:**
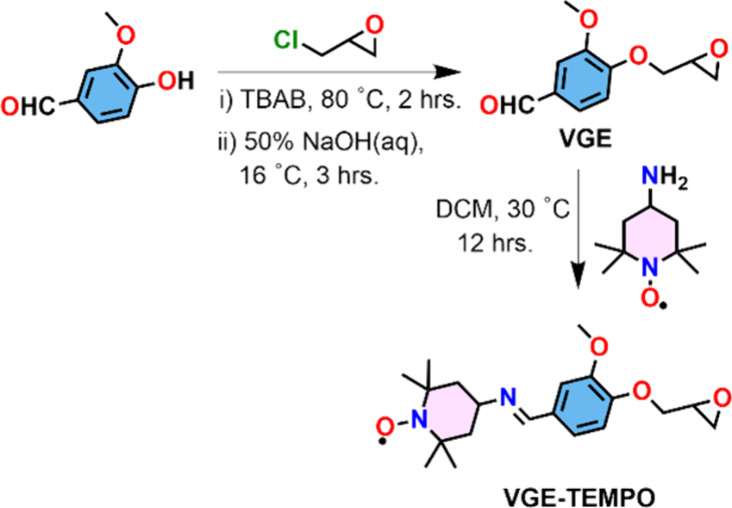
General Synthetic Procedure for VGE and Their VGE-TEMPO Derivatives

Initially, we examined the coupling reaction of VGE with CO_2_ in the presence of the binary catalyst system Co­(salen)­TFA/PPNTFA (TFA = trifluoroacetate). In this instance, at ambient temperature and 3 MPa of CO_2_ pressure, only the cycloaddition product was observed in quantitative yield. The cyclic carbonate product was characterized by ^1^H NMR (Figure S3 in ESI) and crystallized from a dichloromethane/methanol solvent mixture in the monoclinic P2_1_/*n* space group; its structure is shown in Figure S3, and crystallographic parameters are provided in Table S1.

Therefore, we turned our attention to the corresponding coupling reaction of VGE and the more reactive carbonyl sulfide (COS), for in general, these processes have been shown to exclusively afford copolymers. These copolymerization reactions were carried out at 1 MPa COS pressure in the presence of Cr­(salen)­X/PPNX (X = TFA or Cl) at ambient temperature (Scheme 2) with the results listed in Table 1. As noted in Table 1, the molecular weight of the polymer is shown to systematically increase with an increase in the monomer/catalyst ratio while maintaining similar dispersity. This process was found to be completely selective for poly­(monothiocarbonates) (**PMTCs**), as observed by the FTIR, ^1^H NMR (Figure S4), ^13^C NMR (Figure 2), and MALDI-TOF (Figure S5) spectroscopic techniques.^20^

**Scheme 2:**
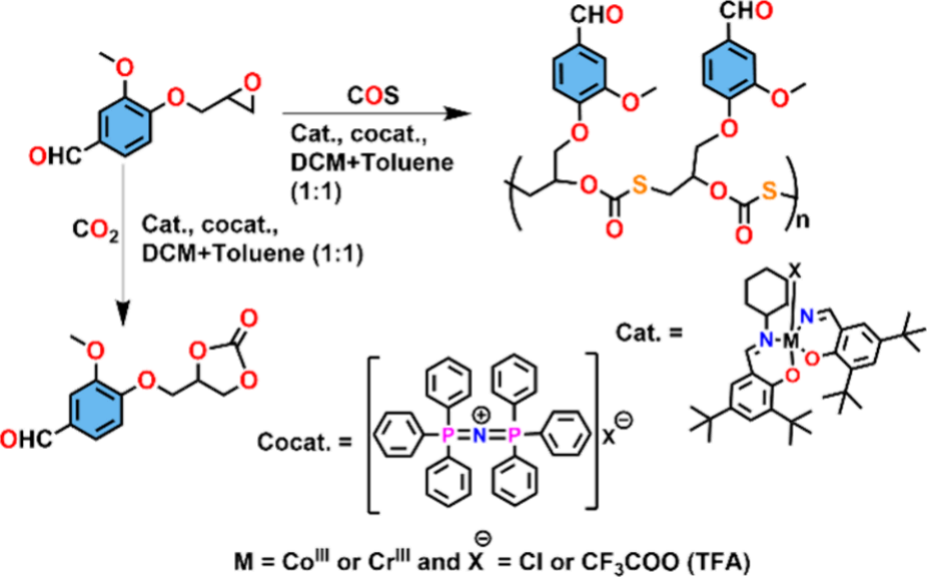
Coupling Reactions of VGE and CO_2_ or COS

**Table 1:** Observed Molecular Weights of Poly­(monothiocarbonates) (**PMTCs**) Using Different Ratios of VGE Monomer^a^

entry	epoxide/cat./cocat.	COS (MPa)	*M*_n_ (kg mol^–1^)	Đ
1^b^	250:1:1	1.0	11.2	1.42
2	250:1:1	1.0	14.8	1.42
3	500:1:1	1.0	30.1	1.41
4	750:1:1	1.0	35.1	1.42
5	1000:1:1	1.0	33.3	1.52

a Reactions were performed with 4 mg of Cr­(salen)­TFA catalyst and solvent 1:1 toluene/DMC in a 15 mL stainless-steel reactor for 12 h.

b Using Cr­(salen)­Cl/PPNCl catalyst.

The resulting **PMTCs** were purified from a dichloromethane solution by adding methanol, a process repeated four times. The molecular weights of polymers were determined in a tetrahydrofuran solvent by gel permeation chromatography (GPC) (Figure 1 and Figure S6). The thermal properties of these **PMTCs** polymers reveal that they are stable up to 200 °C with a glass transition temperature *T*_*g*_ = 64 °C (see TGA and DSC data in Figure S7).

**Figure 1:**
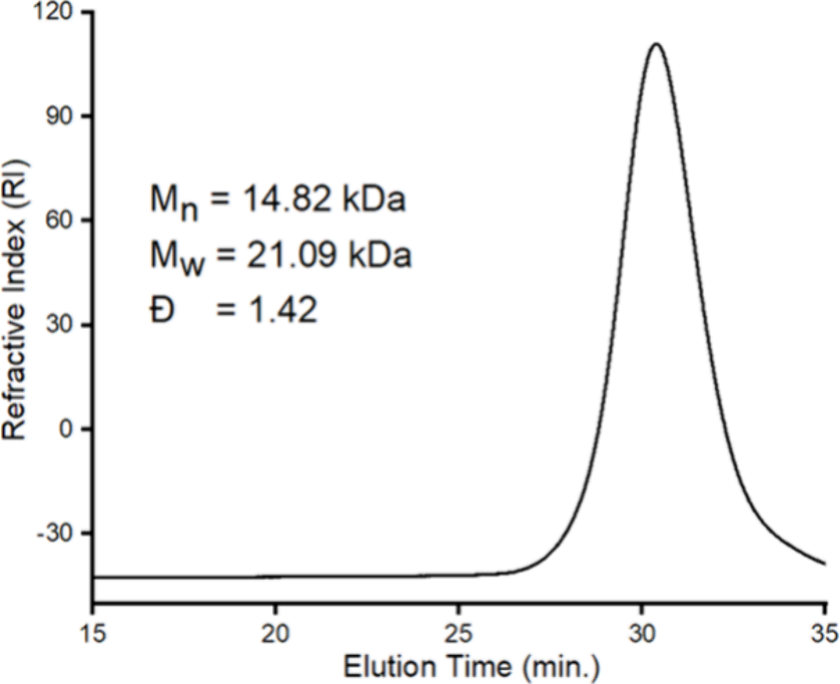
GPC data of PMTC (using Cr­(salen)­TFA catalyst and PPN-TFA cocatalyst recorded in the THF solvent).

Pertinent to the goals of this study, we have employed R-VGE or S-VGE monomers (Figure S8) derived from vanillin and R- or S-epichlorohydrin in the copolymerization reaction with COS, thereby affording an easy pathway for overcoming the challenge of synthesizing regio- and stereoselective polymers with a reactive site for postpolymerization modification. This produces **R-PMTC** and **S-PMTC**, which were characterized (Figures S9 and S10).

As illustrated in Figure 2, the ^13^C NMR of the *atactic* copolymer **PMTC** displayed two closely centered resonances at 169.7 ppm for the carbon center of the monothiocarbonate, whereas, as anticipated, the *isotactic* **R-PMTC** copolymer exhibits a single resonance in this region (Figure 2 and Figure S10). Indeed, these ^13^C NMR spectra further illustrate the absence of any O/S exchange reactions during the polymerization process.

**Figure 2:**
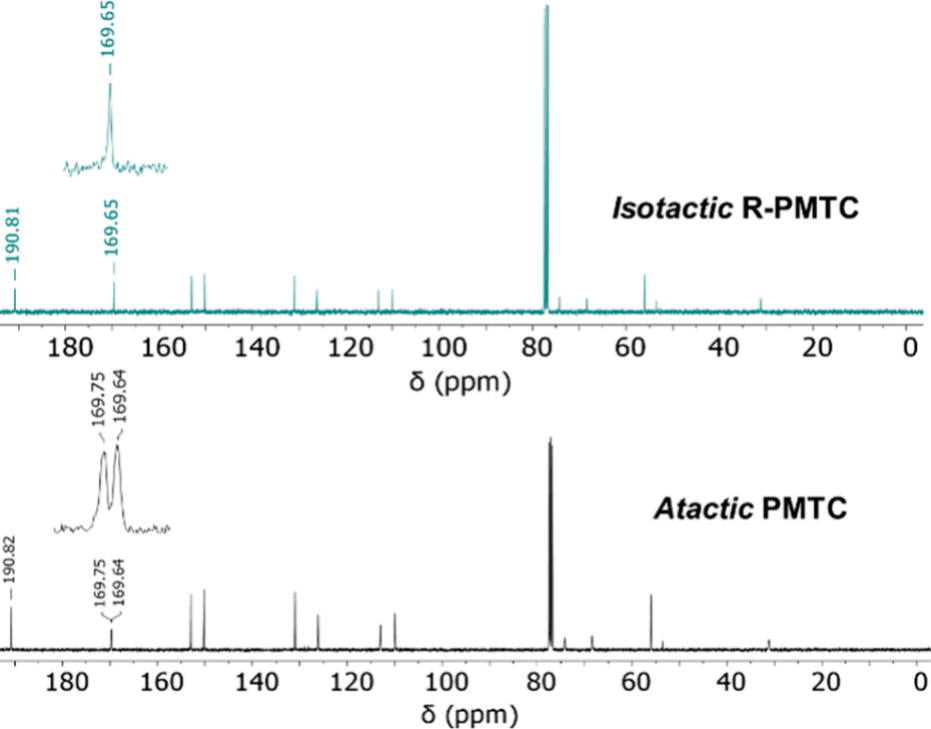
^13^C NMR spectra of *atactic* **PMTC** and *isotactic* **R-PMTC** recorded in CDCl_3_.

Having obtained both *atactic* and *isotactic* versions of these well-characterized copolymers, we have proceeded to synthesize polymers bearing air-stable persistent organic radicals via postpolymerization modification. This was accomplished upon reacting with the aldehyde (−CHO) functional groups available throughout the **PMTC** chain using 4-amino-TEMPO by way of a Schiff base condensation process, as shown in Scheme 3.

**Scheme 3:**
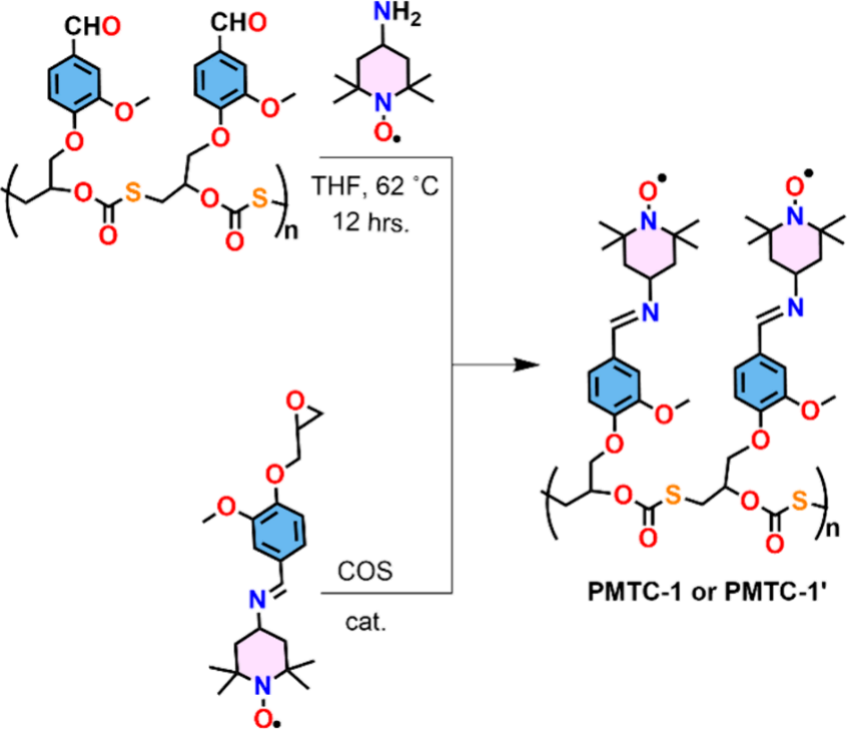
**PMTC-1** or **PMTC-1′** Radical Polymers via Schiff Base Condensation Reaction of **PMTC** with 4-Amino-TEMPO or Copolymerization of COS with VGE-TEMPO Monomer

This Schiff base condensation reaction was easily monitored by infrared and ^1^H NMR spectroscopies. That is, the aldehyde (−CHO) characteristic signal disappears, with the imine (C=N) appearing. Concomitantly, the aldehyde proton signal (−CH−) in the ^1^H NMR spectrum disappears, and the corresponding imine resonance appears (Figure S12). Similarly, the aldehyde (C=O) infrared band in the polymer at 1685 cm^–1^ disappears while imine (C=N) appears at 1641 cm^–1^ (Figure 3).

**Figure 3:**
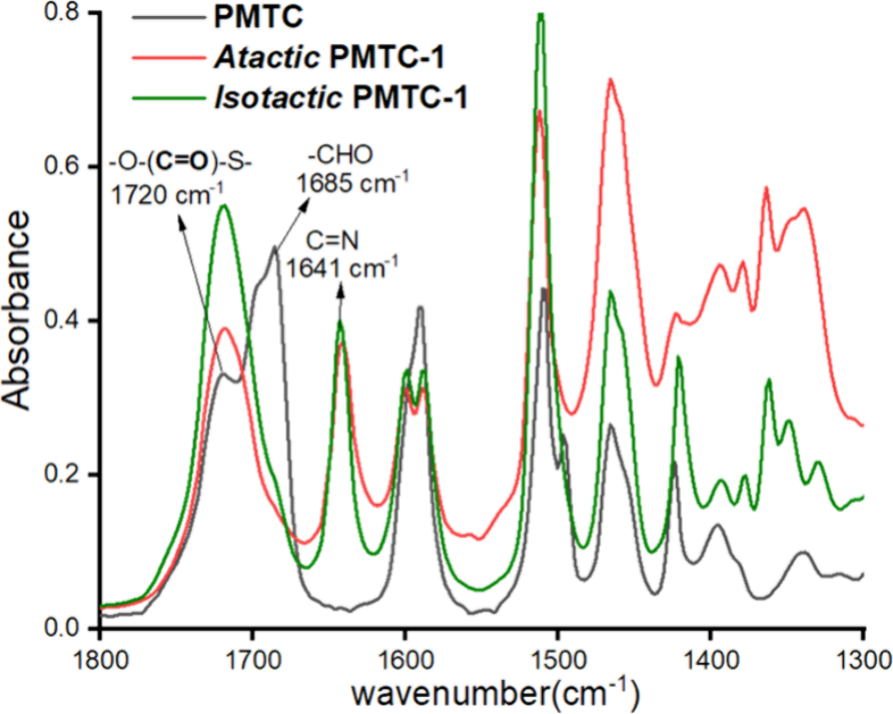
Infrared spectra for the postsynthetic radical polymer **PMTC-1** derived from 4-amino-TEMPO and **PMTC**.

Since polymers containing radicals are widely used for their role in electrochemical and optoelectronic technologies, we have installed air-stable 4-amino-TEMPO radicals into the **PMTC** side chains and produced open-shell *atactic* radical polymers (Scheme 3, **PMTC-1**). Alternatively, the same radical poly­(monothiocarbonate) **PMTC-1′** was also prepared from the VGE-TEMPO radical monomer (Schemes 1 and 3) and COS copolymerization under the same reaction conditions. Here, it is to be noted that the VGE-TEMPO (including R- and S enantiomers) produced low-molecular-weight *M*_n_ = 3–5 kDa (Figures S13–S15 in ESI) radical polymers **PMTC-1′** (**R-PMTC-1′** and **S-PMTC-1′**), and we are yet to establish the conditions to produce high-molecular-weight polymers. However, these low-molecular-weight polymers are suitable candidates to verify whether the nature of magnetic behavior is the same as that of their high-molecular-weight counterparts.

EPR spectroscopic and SQUID magnetometer techniques were employed to characterize and to understand the paramagnetic nature of these radical polymers. The EPR data were collected for the 1 mM solution for each of the polymers and standards (4-amino-TEMPO and VGE-TEMPO) at room temperature (Figure 4 and Figure S16). As for standard 4-amino-TEMPO, the EPR signals for *atactic* **PMTC-1** also show the three maxima with intensities almost 1:1:1. This is due to the hyperfine coupling with ^14^N atoms. This is also observed for all the reported radical polymers. Relative to the standard 4-amino-TEMPO, the radical content in *atactic* **PMTC-1** was found to be ∼78%. This might be due to radical quenching during the reaction and/or purification steps. The EPR signal of *atactic* **PMTC-1** and all polymers in this report was observed to be centered at 3336 G, with estimated g-values of 2.0060 to 2.0064, which are not significantly different from the free electron value of 2.0023.

**Figure 4:**
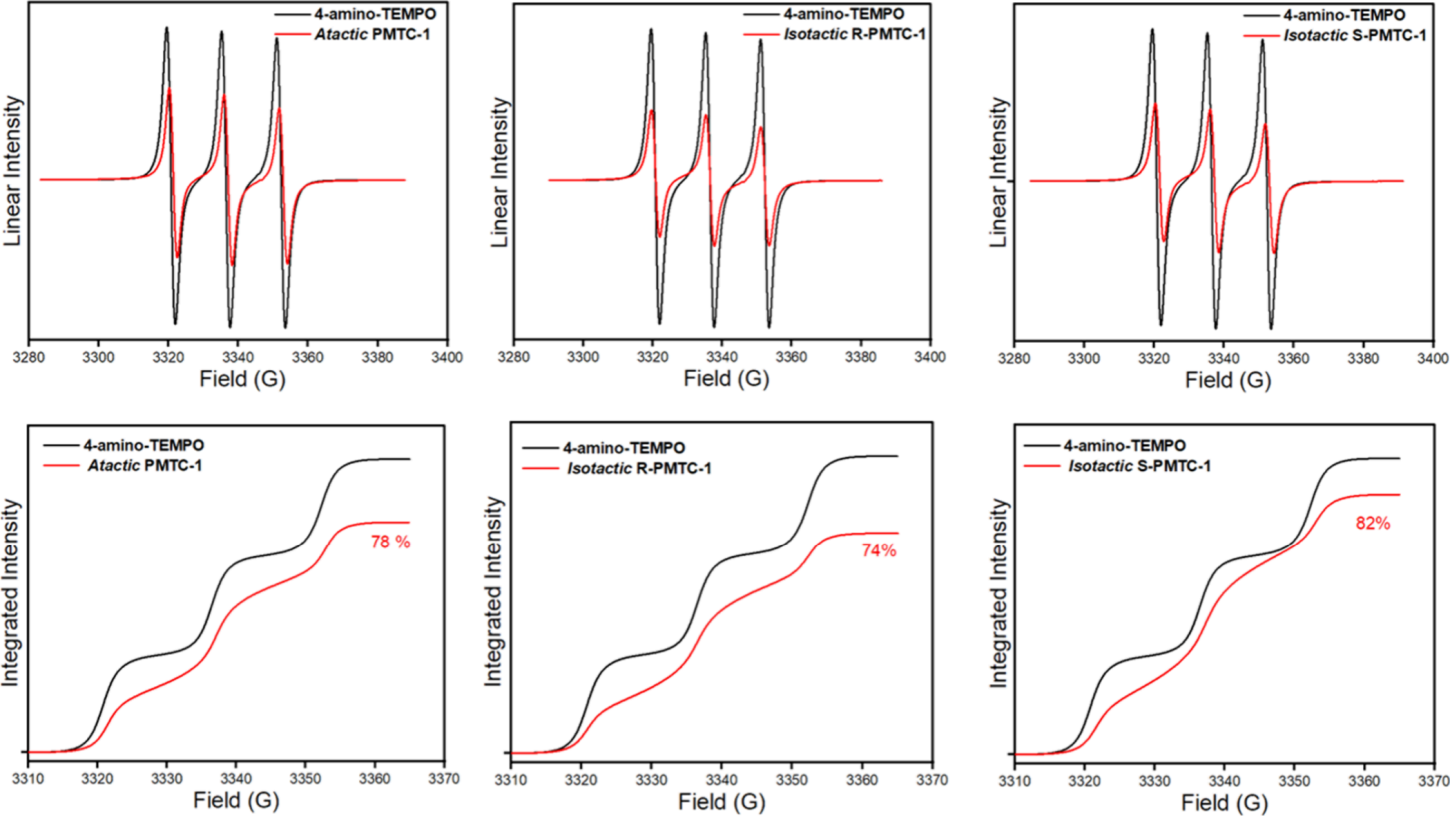
EPR spectra of 4-amino-TEMPO (standard) and *atactic* **PMTC-1**, *isotactic* (**R-PMTC-1**, and **S-PMTC-1**) were recorded for a 1 mM solution in dichloromethane. Integration suggests around 74–82% radical loading in these polymers.

The direct-current magnetic susceptibility data were collected for all these radical polymers in the temperature range of 1.8 to 300 K and under a 1 kOe as well as a 5 kOe applied magnetic field (Figure 5). To confirm any anisotropies associated with structural distortion, we employed zero-field (ZFC) and field-cooled (FC) conditions to collect magnetic susceptibility data. The data were processed and corrected for the diamagnetic contribution using Pascal’s constants.^21^ We did not observe any bifurcation between the ZFC and FC data, suggesting the absence of anisotropy associated with these radical polymers. In general, high temperatures prevent spin alignment or spin ordering in the applied magnetic field, so the materials behave as simple paramagnets. Lowering the temperature decreases the effect of the thermal energy and allows the spin ordering with the applied field, which results in increasing the magnetic susceptibility at lower temperatures. The molar magnetic susceptibility χ_*M*_ values at 300 K for *atactic* **PMTC-1** and *atactic* **PMTC-1′** were found to be 7.92 × 10^–4^ and 9.63 × 10^–4^ cm^3^ mol^–1^, while at 1.8 K, these were 13.86 × 10^–2^ and 14.6 × 10^–2^ cm^3^ mol^–1^, respectively (Table S2 in ESI). The room-temperature χ_*M*_ value was found to be lower than the theoretical value of 1.25 × 10^–3^ cm^3^ mol^–1^ for an unpaired electron (g = 2.0 and S = ^1^/_2_). The observed low-susceptibility value may be due to the dominant antiferromagnetic interaction between radicals, and/or also to the lower radical concentration in the side chain (confirmed from EPR). To determine the nature of magnetic interaction between radicals, we followed the Curie–Weiss law (eq 1):

$$\chi_{M}=\frac{C}{T-\theta }$$ 1

$$C=\frac{N_{A}\mu_{eff}^{2}}{3k_{B}}$$

where *C* is the Curie constant (unpaired spins per mole of paramagnetic material) and θ is the Curie–Weiss constant. We use eq 1 to fit the χ_*M*_^–1^ = *f*(T) plot from 300 to 1.8 K (Figure 5).

**Figure 5:**
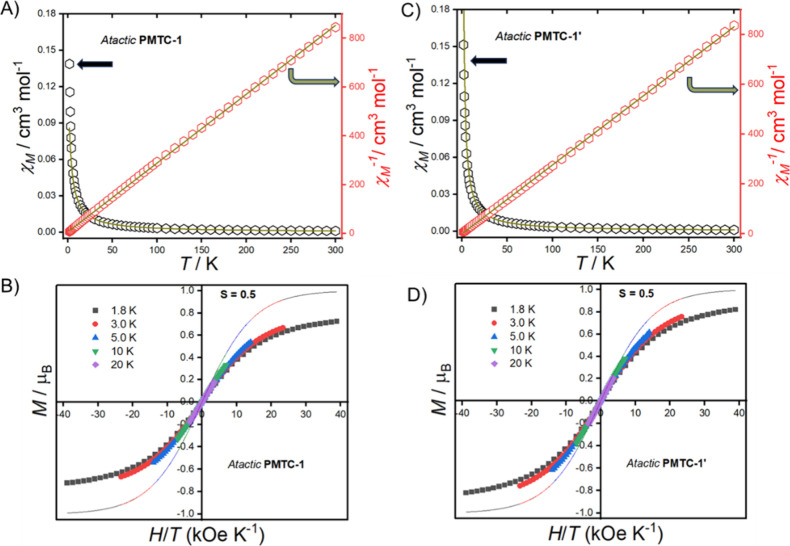
Temperature-dependent direct-current magnetic susceptibilities under 1 kOe magnetic field (panels A&B) and field-dependent magnetization (panels C&D) data for *atactic* **PMTC-1** and **PMTC-1′**, respectively. The magnetic susceptibility data were plotted as χ_*M*_^–1^ = *f*(T) and χ_*M*_ = *f*(T). Field-dependent magnetization data at the indicated temperatures were plotted as *M* = *f*(H/T). The solid lines or curves represent the fitted data.

The best fit of 1 kOe data (Figure 5) yielded a Curie constant *C* = 0.35 and θ = −20.3 K (for *atactic* **PMTC-1**) and *C* = 0.36 and θ = −5.4 K (for *atactic* **PMTC-1′**), while the best fit of 5 kOe data yielded *C* = 0.25 and θ = −30.5 K (for *atactic* **PMTC-1**) and *C* = 0.29 and θ = −12.2 K (for *atactic* **PMTC-1′**), which indicates dominant antiferromagnetic interactions in these *atactic* polymers. Here, it is to be noted that the racemic (±)-epichlorohydrin used to synthesize the VGE monomer, and hence the poly­(monothiocarbonates), will fall in the category of “*atactic*” polymer (also confirmed from ^13^C NMR, Figure 2). In this *atactic* **PMTC**, the pendant aldehyde groups are expected to be statistically oriented as shown in Figure 6, and hence, the installed radicals are also oriented similarly in *atactic* **PMTC-1** (or in *atactic* **PMTC-1′**).

**Figure 6:**
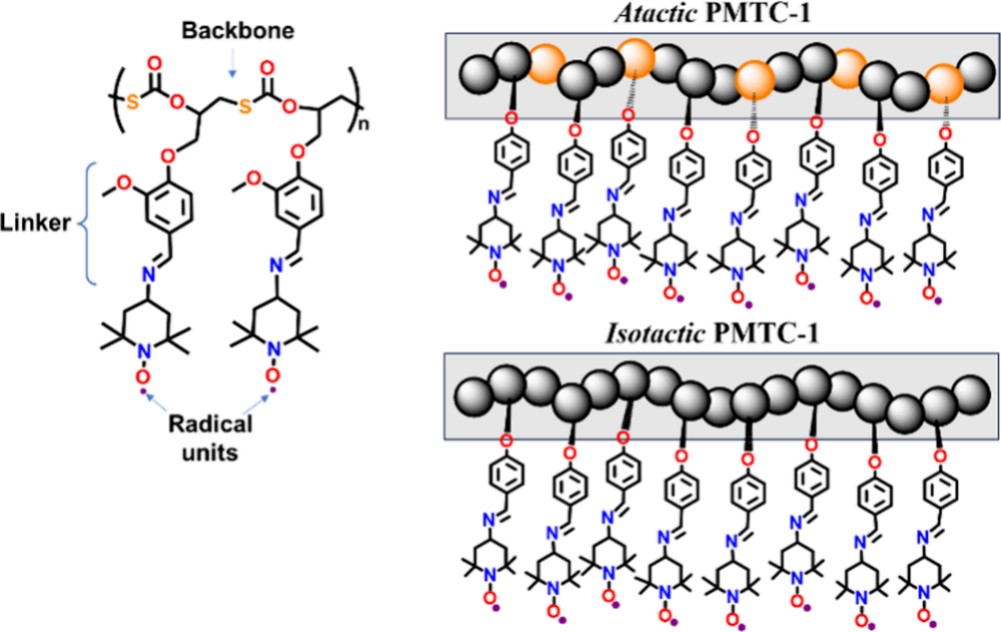
Radical functionalized *atactic* and *isotactic* **PMTCs**.

In our previous studies, we have shown that *isotactic* poly­(monothiocarbonates) containing TEMPO radical units at ambient temperature exhibited greater conductivity (*∼*10^–4^ S cm^–1^) than their *atactic* analog (*∼*10^–7^ S cm^–1^), where the electrical conductivities of these radical polymers were measured as solid-state films deposited on gold electrodes.^13^ In this report, we were interested in understanding the nature of magnetic interactions between radicals that are expected to be highly ordered and arranged regularly in an *isotactic* **R-PMTC-1** or **S**-**PMTC-1.**

Therefore, we installed 4-amino-TEMPO and produced *isotactic* **R-PMTC-1** and **S-PMTC-1** radical polymers (Figures S12 and S15), then performed magnetic measurements under the same conditions as for *atactic* **PMTC-1** and **PMTC-1′** polymers and observed the room-temperature χ_*M*_ values of 8.75 × 10^–4^ and 8.23 × 10^–4^ cm^3^ mol^–1^, while at 1.8 K, these are 12.99 × 10^–2^ and 13.41 × 10^–2^ cm^3^ mol^–1^, respectively (Table S2 in ESI). The nature of magnetic interactions is found to be dominant ferromagnetic, as estimated from the χ_*M*_^–1^ = *f*(T) curve fitting (Figure 8 and Table S3), which yields *C* = 0.37 and θ = +17.6 K (for *isotactic* **R-PMTC-1**) and *C* = 0.37 and θ = +16.9 K (*isotactic* **S-PMTC-1**), respectively, under a 1 kOe magnetic field. A similar response was also observed for low-molecular-weight *isotactic* **R-PMTC-1′** and **S-PMTC-1′** radical polymers (Figure 8 and Table S3). In comparison to *atactic* polymers (**PMTC-1** and **PMTC-1′**), which show dominant antiferromagnetic behavior, the *isotactic* or stereoregular **R-PMTC-1**, **S-PMTC-1, RPMTC-1′**, and **S-PMTC-1′** radical polymers show dominant ferromagnetic behavior under a 1 kOe magnetic field. Under 5 kOe of applied magnetic field, the best fit of the χ_M_^–1^ vs *T* curves (Figure S17) for all *atactic* and *isotactic* radical polymers shows dominant antiferromagnetic behaviors. To better understand the magnetic response of *atactic* and *isotactic* radical polymers under various applied magnetic fields, we have collected data under the applied fields of 0.5 to 5 kOe with an interval of 0.5 kOe, and the θ values are reported in Table S3. The strength of the ferromagnetic interactions in *isotactic* polymers decreases with increasing magnetic field, while at a certain magnetic field, the interaction switches to antiferromagnetic. For instance, the strength of ferromagnetic interaction decreases from θ = +25.3 K to θ = +0.1 K and then switches to antiferromagnetic θ = −4.3 K at 3.0 kOe and reaches −17.4 K at 5 kOe for *isotactic* **R-PMTC-1′** (Table S3). A regular pattern of magnetic switching with increasing applied magnetic field was observed for *isotactic* polymers, while *atactic* polymers are nonswitchable and or exhibit irregular behaviors (also see the graphs at the bottom of Table S3).

This is due to the highly ordered arrangement of TEMPO radicals in *isotactic* **R-PMTC-1** (or **R-PMTC-1′**) and **S-PMTC-1** (or **S-PMTC-1′**) compared to the irregular arrangement in the *atactic* analogue. For polymeric materials, it is not surprising that the structural changes can tune the nature of the dominant magnetic interaction, i.e., from antiferromagnetic to ferromagnetic or vice versa.^22^ It should be noted here that the orientation of radicals from statistical to a regular way does not necessarily promote the dominating ferromagnetic exchange in all examples. We observed that the molecular weight of polymers affects the strength, but the nature of magnetic interaction remains the same for these reported *atactic* and *isotactic* polymers. The nature and strength of magnetic interactions also depend on factors such as distance, angle, and others. For this purpose, we performed magnetic measurements under similar conditions on our previously reported *atactic* and *isotactic* radical poly­(monothiocarbonates)^13^ (hereafter referred to as *atactic* **GTEMPO-PMTC** and *isotactic* **R-GTEMPO-PMTC**; Figure 7), derived from racemic GTEMPO and enantiomer R-GTEMPO monomers, respectively. These polymers have the same poly­(monothiocarbonate) backbones (*atactic* or *isotactic*) and open-shell TEMPO radical units throughout the side chain as in the present case of TEMPO-installed **PMTCs**. Since they are derived from different monomers, structurally, both kinds of polymers (**PMTC-1** and **GTEMPO-PMTC**) differ in the distance of the radicals from the backbone, depending on the linker that links radical units to the backbone. In the case of **GTEMPO-PMTCs**, radical units are closer to the backbone as compared to **TEMPO-**installed **PMTC-1** (Figures 6 and 7). For *atactic* **GTEMPO-PMTC**, the molar magnetic susceptibility χ_*M*_ at 300 K was found to be 8.67 × 10^–4^ cm^3^ mol^–1^, and at 1.8 K, it was 13.8 × 10^–2^ cm^3^ mol^–1^, while for *isotactic* **R-GTEMPO-PMTC**, the room-temperature value was found to be 12.0 × 10^–4^ cm^3^ mol^–1^, and at 1.8 K, this was 15.3 × 10^–2^ cm^3^ mol^–1^.

**Figure 7:**
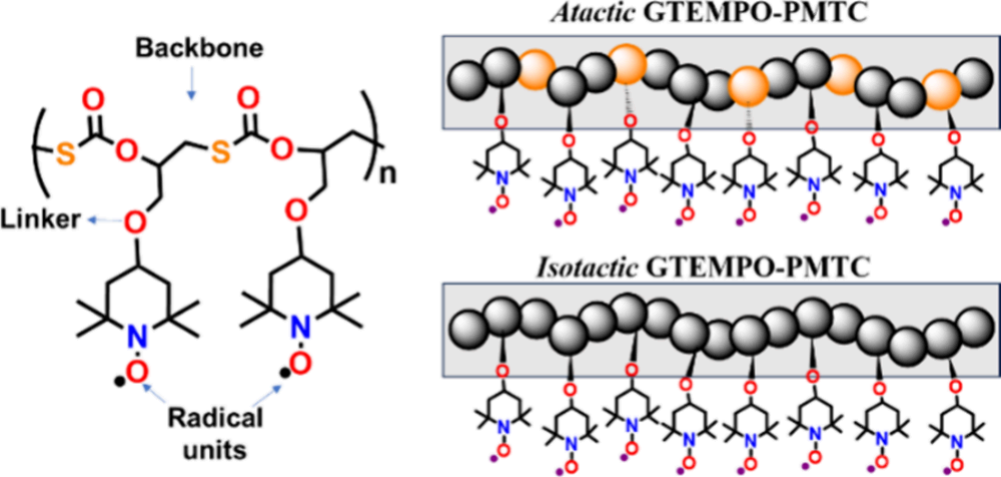
*Atactic* and *isotactic* **GTEMPO**-**PMTCs**.

The best fit of the χ_*M*_^–1^ = *f*(T) plot yielded θ = −18.4 K with *C* = 0.29 (from 1 kOe data) and θ = −28.4 K with *C* = 0.27 (from 5 kOe data) for *atactic,* while for *isotactic* θ = +1.68 K with *C* = 0.42 (from 1 kOe data) and θ = −10.5 K with C = 0.37 (from 5 kOe data). This observation indicates that the magnetic exchange in *atactic* **GTEMPO-PMTC** polymer is stronger and dominantly antiferromagnetic, while in stereoregular *isotactic* **R-GTEMPO-PMTC**, this is weaker but ferromagnetic in nature. The difference in the magnetic exchange coupling observed in *atactic* and *isotactic* polymers is attributed again to their backbone’s stereochemistry. The magnetic behaviour under 1 kOe and 5 kOe magnetic fields for *atactic* and *isotactic* radical polymers is shown in Figure 9.

These results suggest the role of tacticity in controlling the orientation of radical units and hence the magnetic behavior of radical polymers, which can be seen under applied magnetic fields. The *tacticity-controlled* magnetic behavior of radical polymers, where the strength and sign of the magnetic exchange can be tuned, is a suitable candidate for tunable magnetoresistance (MR) response studies, along with other technological applications. After the observation of tunable magnetic behavior of these radical polymers, in our next move, we intend to apply these polymers for further applications in various electronic devices.

The field-dependent magnetization measurements were performed at temperatures 1.8 to 20 K on all the *atactic* (**PMTC-1**, **PMTC-1′**, and **GTEMPO-PMTC**) and *isotactic* **R-PMTC-1**, **S-PMTC-1**, **R-PMTC-1′**, **S-PMTC-1′**, and **R-GTEMPO-PMTC**) samples under an applied magnetic field sweep of −70 to +70 kOe. At high magnetic fields, the spins are expected to align with the field, and the magnetization increases linearly, reaching saturation *M*_sat_ at ±70 kOe. This behavior can be modeled using the Brillouin function B_s_(*x*), assuming no spin–spin interactions.

$$M=M_{\mathrm{sat}}B_{s}\left(x\right)$$ 2

The Brillouin function B_s_(*x*) can be defined as

$$B_{s}\left(x\right)=\frac{2S+1}{2S}\coth\left(\frac{2S+1}{2S}.x\right)-\frac{1}{2S}\coth\left(\frac{1}{2S}.x\right)$$ 3

where

$$x=\frac{{gS\mu }_{B}H}{k_{B}T}$$

The observed magnetization saturation *M*_sat_ values of 0.72 μ_*B*_ (for **PMTC-1**), 0.73 μ_*B*_, (for **R-PMTC-1**), and 0.75 μ_*B*_ (for **S-PMTC-1**), respectively (Table S2), were found to be lower compared to the theoretical value of 1.0 μ_*B*_ for a single unpaired electron. In contrast, the *atactic* **PMTC-1′** and *isotactic* **R-PMTC-1′** and **S-PMTC-1′** polymers show values of 0.82 μ_*B*_, 0.92 μ_*B*_, and 0.87 μ_*B,*_ respectively, which are higher compared to the postradical functionalized polymers (Table S2). Our EPR studies on these polymers show that the radical content is ∼74–92% (Figure S16), which can be correlated to the observed low values of *M*_sat_ compared to the theoretical value.

In the cases of *isotactic* radical polymers, the magnetization saturation values are comparatively higher due to ferromagnetic interactions or weaker antiferromagnetic interactions. We fitted the magnetization curves to the Brillouin function B_s_(*x*) with spin *S* = 1/2. The magnetization curve fittings were excellent for all the samples at temperatures ≥10 K (Figures 5 and 8 and Figure S18), while a diversion was observed below 10 K.

**Figure 8:**
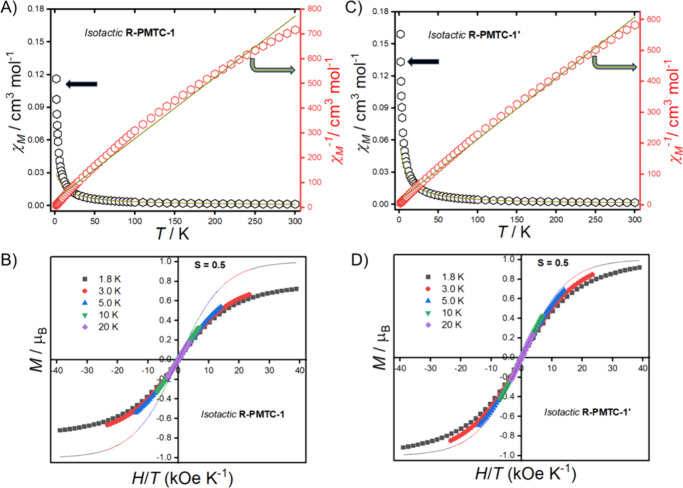
Temperature-dependent direct-current magnetic susceptibility measured under 1 kOe magnetic field and field-dependent magnetization data for *isotactic* **R-PMTC-1** (A,B) and *isotactic* **R-PMTC-1′** (C,D). The magnetic susceptibility was plotted as χ_*M*_^–1^ = *f*(T) and χ_*M*_ = *f*(T). Field-dependent magnetization data at the indicated temperatures were plotted as *M* = *f*(H/T). The solid lines or curves represent the fitted data.

**Figure 9:**
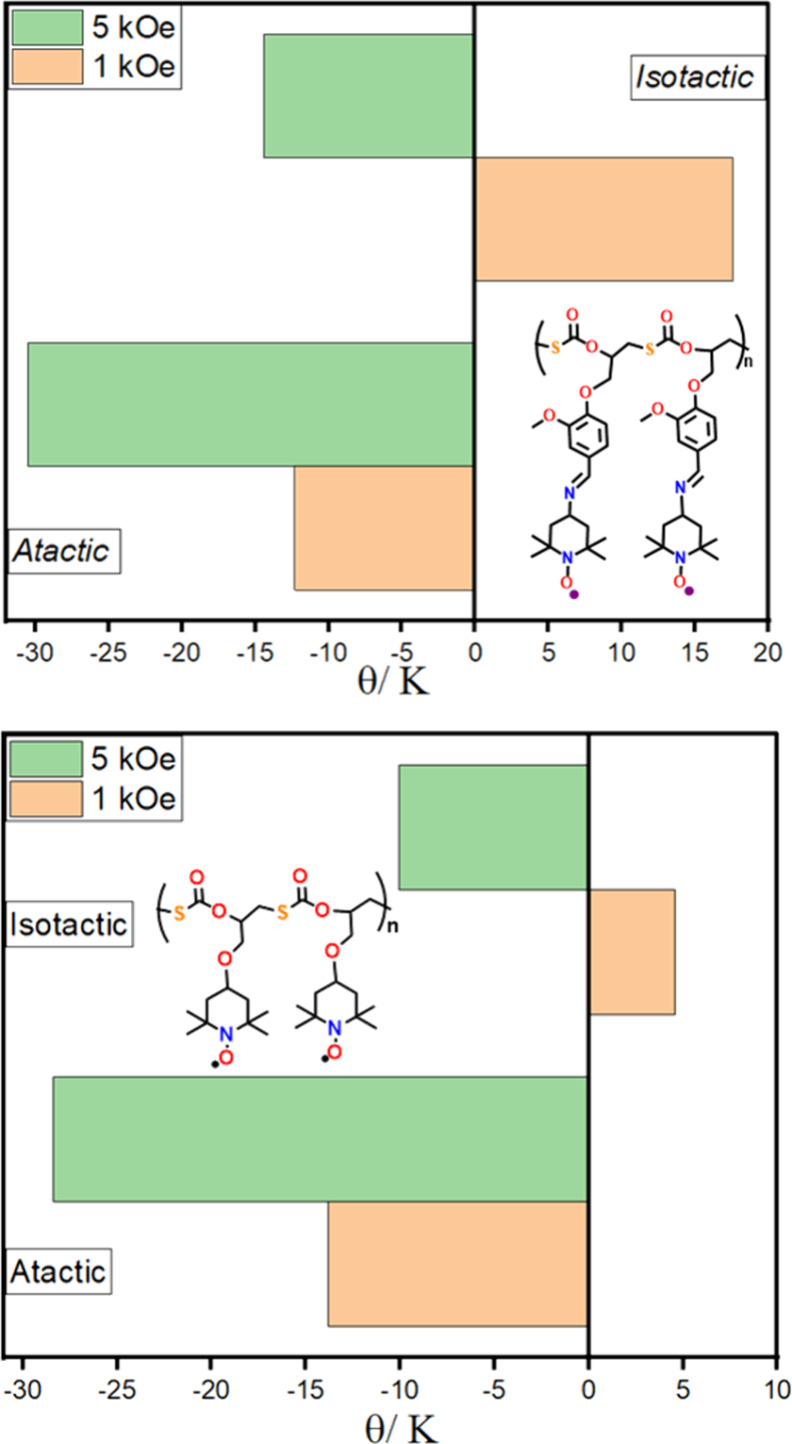
Graphical presentation of magnetic interactions in *atactic* and *isotactic* polymers under 1 and 5 kOe applied magnetic field.

To investigate the reasons and rationalize the preferred nature of the dominating magnetic interactions in *atactic* **PMTC-1** and *isotactic* **R-PMTC-1** and **S-PMTC-1** polymers, we performed theoretical studies on the optimized models of the respective polymers (see Section 4).

In further studies designed to better understand the redox nature of all these radical polymers, we employed cyclic voltammetry in a dichloromethane solvent under an argon atmosphere, using 0.1 M [tBu_4_N]­[PF_6_] as the supporting electrolyte, referenced to Fe^+/0^ (*E*_1/2_ = 0.0 V). The appearance of the reversible oxidation in the voltage range of +0.30 to +0.41 V confirms the redox nature (Figure S19) of these radical polymers. We observed a slight but not significant difference in the redox potential of 4-amino-TEMPO compared with radical polymers.

## 4 Computational Studies

To rationalize the experimentally observed nature of magnetic interactions in *isotactic* **R-PMTC-1**, we modeled the dimeric structures, which consist of two TEMPO units. Based on the *isotactic* projection, we kept these TEMPO units with the same chirality along the thiocarbonate backbone and optimized the structure. In the optimized structure of **R-PMTC-1** **^mod^**, π–π interaction is found between the neighboring phenyl rings of vanillin. This brings the two TEMPO units into close contact and aligns them in an orthogonal position, with an interplanar angle of 77.3°, which in turn results in radicals (N_TEMPO_···N_TEMPO_) being closer together by 6.34 Å (Figure 10B) in the range that allows effective magnetic communication. We performed BS-DFT (broken symmetry density functional theory) calculations on the optimized **R-PMTC-1** **^mod^** using the UB97D functional, utilizing the Gaussian 16.0 software platform. The details of the computational method employed for optimization and BS-DFT calculations are provided in the ESI. The spin density plots suggest that most of the spins are localized on nitrogen and oxygen (Figure 11). The BS-DFT calculations are widely used in estimating the magnetic exchange between metals (transition and lanthanide metal ions) and radicals.^23–28^ To estimate the *J* value, we used the Yamaguchi spin-projection formula (eq 4), which yields *J* = +2.83 K and rationalizes our experimental finding of a weak ferromagnetic interaction between TEMPO radicals in the *isotactic* **R-PMTC-1** polymer.

**Figure 10:**
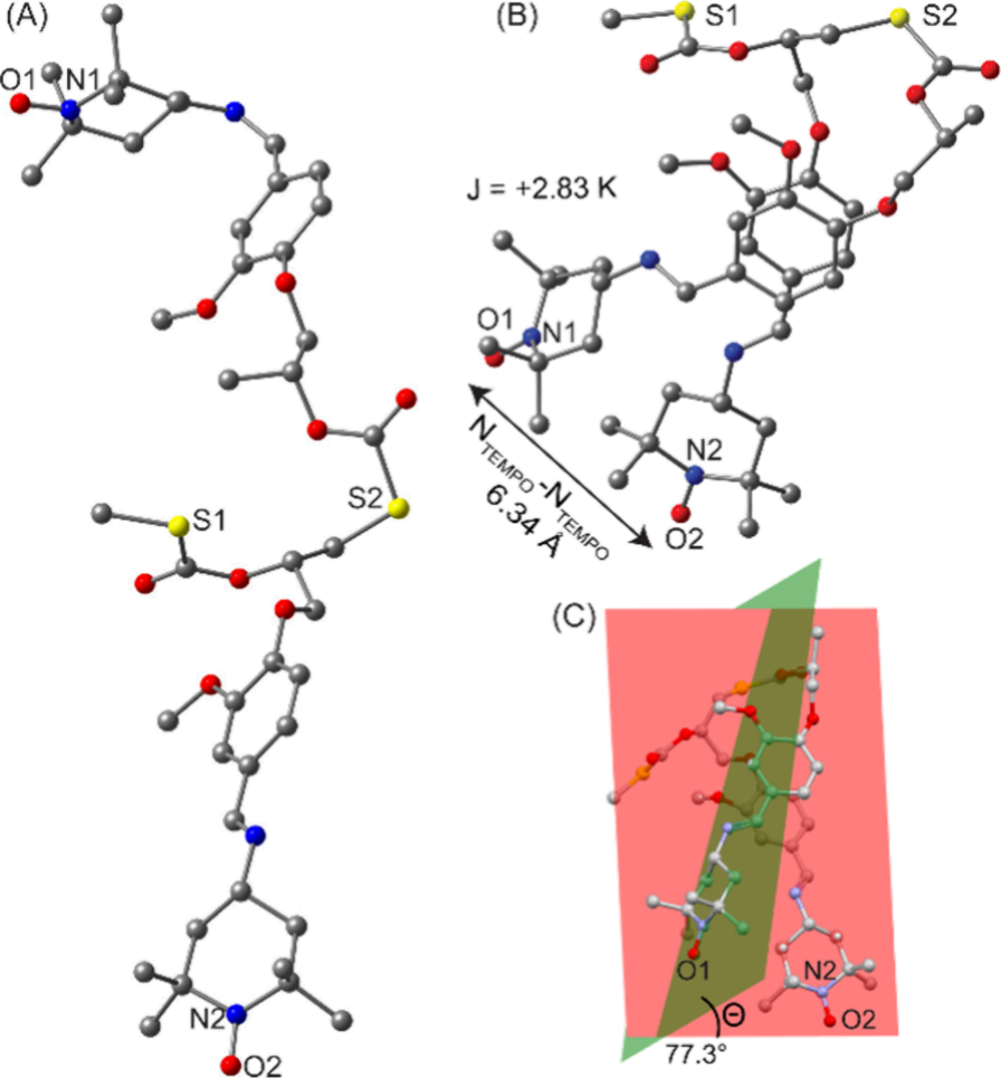
UB97*D*/6–311+G** optimized geometries (A) for *atactic* **PMTC-1** ^mod^ oligomer and (B) for *isotactic* **R-PMTC-1** ^mod^ oligomer. (C) Diagram illustrating the angle Θ between the least-squares planes of the C_5_NO TEMPO core in the **R-PMTC-1** ^mod^ oligomer.

**Figure 11:**
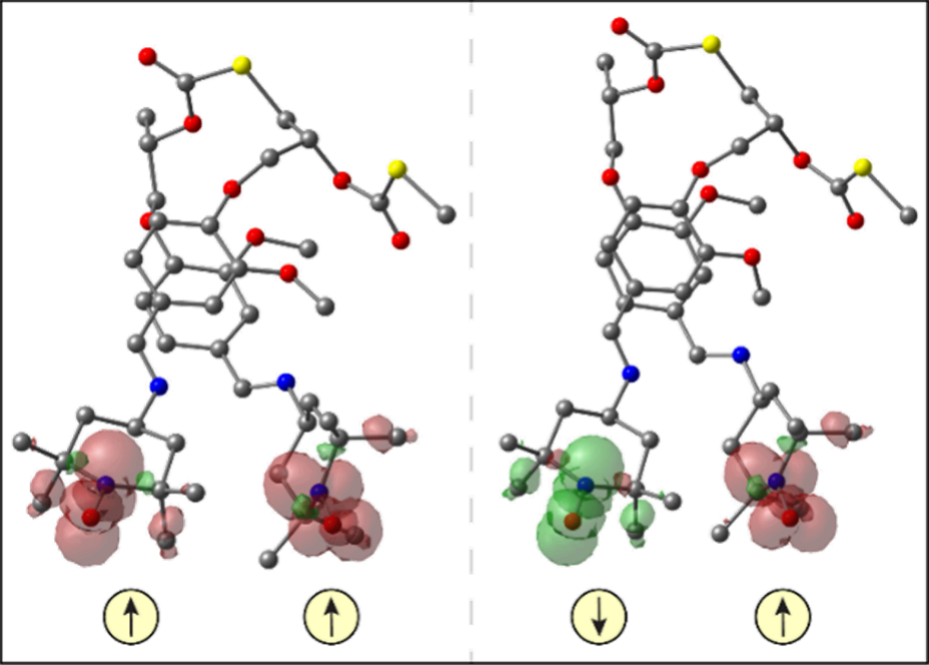
Spin density plots of *isotactic* oligomer (isovalue = 0.002).

We have also performed computational analysis on the modeled structure of *isotactic* **S-PMTC-1**. The (N_TEMPO_ ···N_TEMPO_) distance is 5.49 Å, with an interplanar angle of 15.4°. The calculated coupling constant between the two TEMPO units is 1.07 K, which is consistent with the expected dependence of magnetic interaction on the interplanar angle.

Similarly, to rationalize the observed experimental findings of a dominating antiferromagnetic interaction in the *atactic* **PMTC-1** polymer, we modeled **PMTC-1** **^mod^** by projecting two TEMPO units opposite in orientations on the backbone and optimizing the structure. In the optimized structure of **PMTC-1** **^mod^**, we observed the large N_TEMPO_···N_TEMPO_ distance of 24 Å between the two TEMPO units, which is too large for any significant magnetic interaction.

Since the tacticity is confined to stereochemistry at the backbone stereocenters, not by the visual placement of the group along the polymer chain, long side groups having different stereo configurations can bend around and end up on the same side of the chain. To address the polymer **PMTC-1** **^mod^** structure better and related conformational flexibility and magnetic properties, we performed DFT calculations on an oligomer containing four TEMPO units. We have defined the stereochemistry of the carbon atoms bearing the side groups R, S, R, S, starting from the −S–CH_3_ end and labeled the attached TEMPO units as 1, 2, 3, and 4 (Figure 12A). At the B3LYP level of calculation, we can clearly visualize the TEMPO groups and observe that they are positioned far apart, with the closest N_TEMPO_···N_TEMPO_ distance exceeding 10 Å (Figure S20). After inclusion of dispersion correction with UB97D functional, which has been found good for through-space interactions,^29^ the TEMPO units come closer, and the N_TEMPO_···N_TEMPO_ distance between 1 and 2, 2–3, and 1–3 is reduced to 5.80, 6.99, and 6.06 Å, respectively (Figure 12B). We have calculated the magnetic coupling constant between these units using the Yamaguchi formula. The magnetic exchange between 1 and 2 units was found to be antiferromagnetic (*J* = −15 K). It is important to note that the carbon-bearing 1 and 2 groups adopt different conformations, an opposite scenario than the isotactic dimer, and the extent of coupling is greater when compared with the *isotactic* dimer, which shows a much smaller exchange (*J* = +2.83 K). The magnetic exchange between 1 and 3 was found to be ferromagnetic (*J* = +2.48 K) as they possess a similar conformation to that of an *isotactic* dimer. The magnetic exchange between 2 and 3 was found to be very weak with a *J* value of 0.40 K. We have also examined **GTEMPO-PMTC**, which revealed that the *isotactic* dimer shows negligible magnetic exchange (−0.37 K); however, in the case of *atactic*, its value increased to −3.29 K (Figure S21). It is worthwhile comparing the interplanar angle between these units. The interplanar angle between 1,2, and 2,3 and 1,3 TEMPO units was found to be 34.64°, 68.61°, and 85.88°, respectively. In fact, the nature of magnetic interaction depends strongly on the relative orientation of the TEMPO units. When they are orthogonal (interplanar angle >65°), the magnetic interaction is ferromagnetic; however, if they are nearly planar, the interaction is antiferromagnetic. Such dependence of magnetic exchange on the relative orientation of spin centers has also been observed in discrete metal complexes bearing ligand-centered radicals.^30,31^

$$J=\frac{E_{U-\mathrm{BS}}^{\mathrm{LS}}-E_{U}^{\mathrm{HS}}}{{\left\langle \hat{S^{2}}\right\rangle }_{U}^{\mathrm{HS}}-{\left\langle \hat{S^{2}}\right\rangle }_{U}^{\mathrm{LS}}}$$ 4

**Figure 12:**
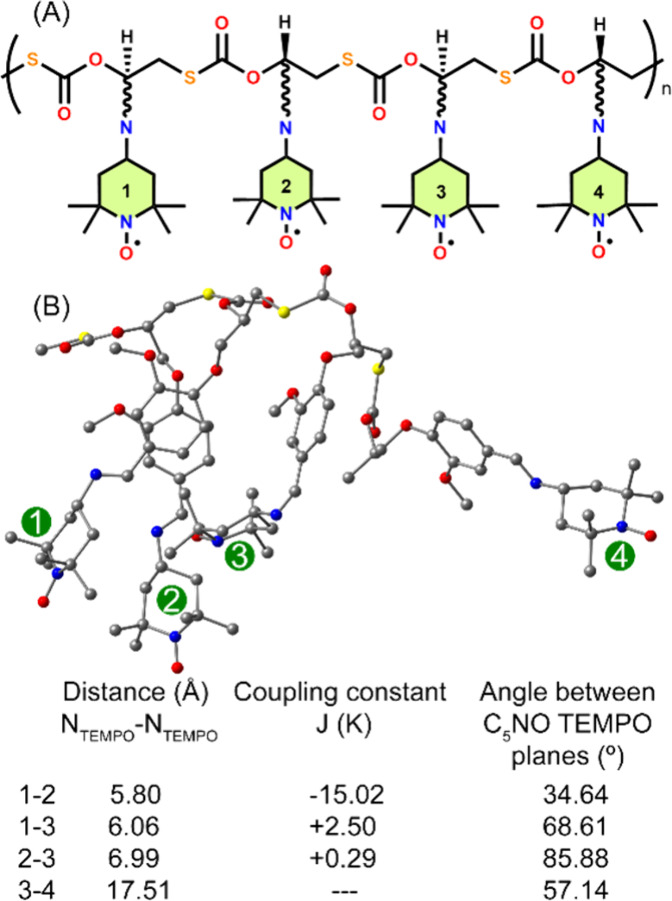
(A) Schematic representation of the modeled oligomer (tetramer). Hydrogen atoms in the scheme are used to show the stereochemistry of carbon-bearing side groups. (B) UB97D optimized geometry of a tetramer of **PMTC-1** ^mod^ along with N_TEMPO_···N_TEMPO_ distances and coupling constant *J*.

## 5 Conclusions

In summary, we have synthesized and utilized epoxide monomers, vanillin glycidyl ether (racemic VGE, and its enantiomers R-VGE and S-VGE), derived from biosourced vanillin and epichlorohydrin (racemic or R- or S- enantiomers), for the production of respective poly­(monothiocarbonates) (**PMTCs**) via catalytic copolymerization with COS. Furthermore, we utilized the pendant formaldehyde groups on these polymers’ side chains for postsynthetic modification via Schiff base condensation with 4-amino-TEMPO radicals. The TEMPO-installed radical polymers (**PMTCs-1**) were studied in detail for their redox and magnetic behaviors, and it was noted that *tacticity* controlled the nature of magnetic exchange coupling between the radical units. We also verified the similar magnetic behavior from the same but low-molecular-weight *atactic* and *isotactic* radical polymers (**PMTCs-1′**), produced from preinstalled radical VGE-TEMPO monomers. In another example of a radical polymer derived from a GTEMPO monomer, a similar scenario was observed. This indicates that changing the *tacticity* can tune or modulate the overall magnetic behavior along the polymeric chain that can be utilized for tunable magnetoresistance and other studies for device applications.

## Supplementary Material



## Notes

Supporting structural parameters, spectroscopic, and magnetic data; X-ray data (PDF)
