# Supplementary material for: Role of Stereochemistry in Controlling Magnetic Behavior in Polymeric Materials
Source: J Am Chem Soc. 2026 May 18;148(21):21704–13. doi: 10.1021/jacs.6c01683 (PMC13244446; doi:10.1021/jacs.6c01683)
Supplement: Supplementary file 1 [file ja6c01683_si_001.pdf]

Supporting Information

Role of Stereochemistry in Controlling Magnetic Behavior in Polymeric Materials

Naushad Ahmed,[a] Akhil Kumar Singh,[a] Mani Sengoden,[a] Marcetta Y. Darensbourg, and Donald J. Darensbourg*[a]

[a] *Department of Chemistry, Texas A&M University, College Station, Texas 77843, United States*

Email: djdarens@chem.tamu.edu

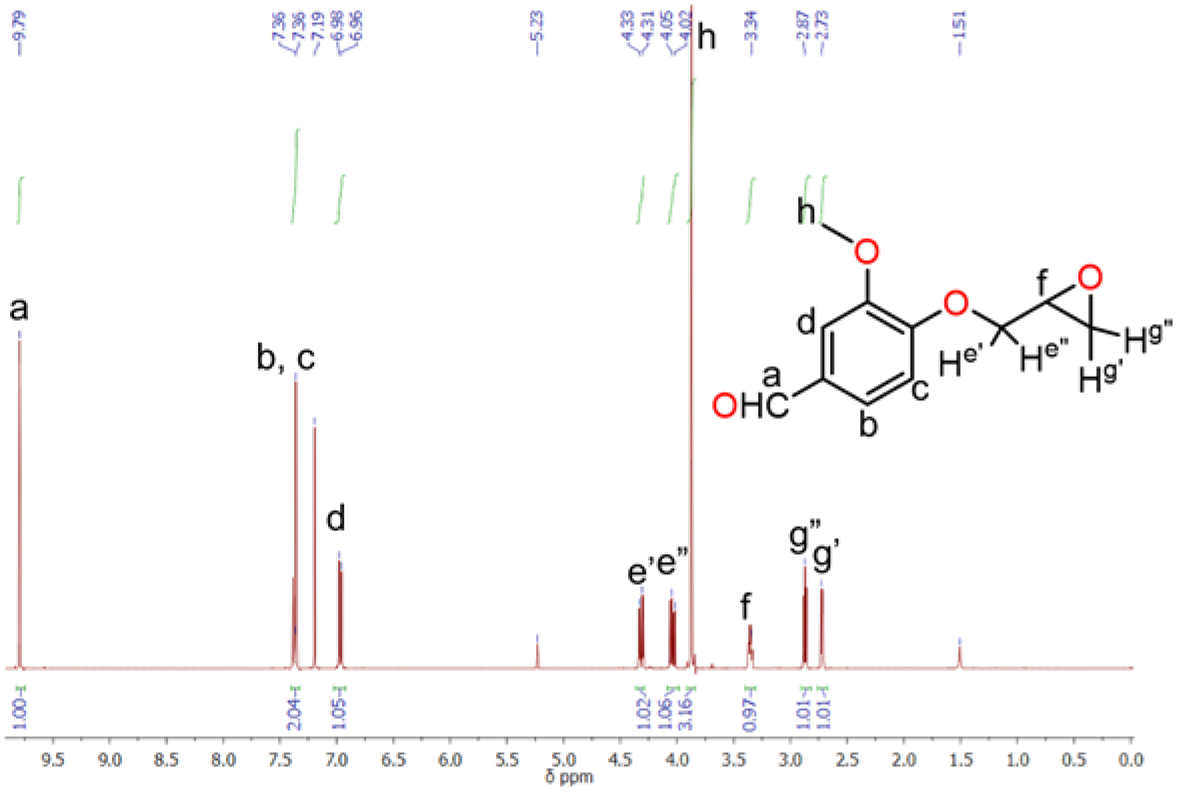

Figure S1. ^{1}H NMR spectra of VGE monomer in $CDCl_3$.

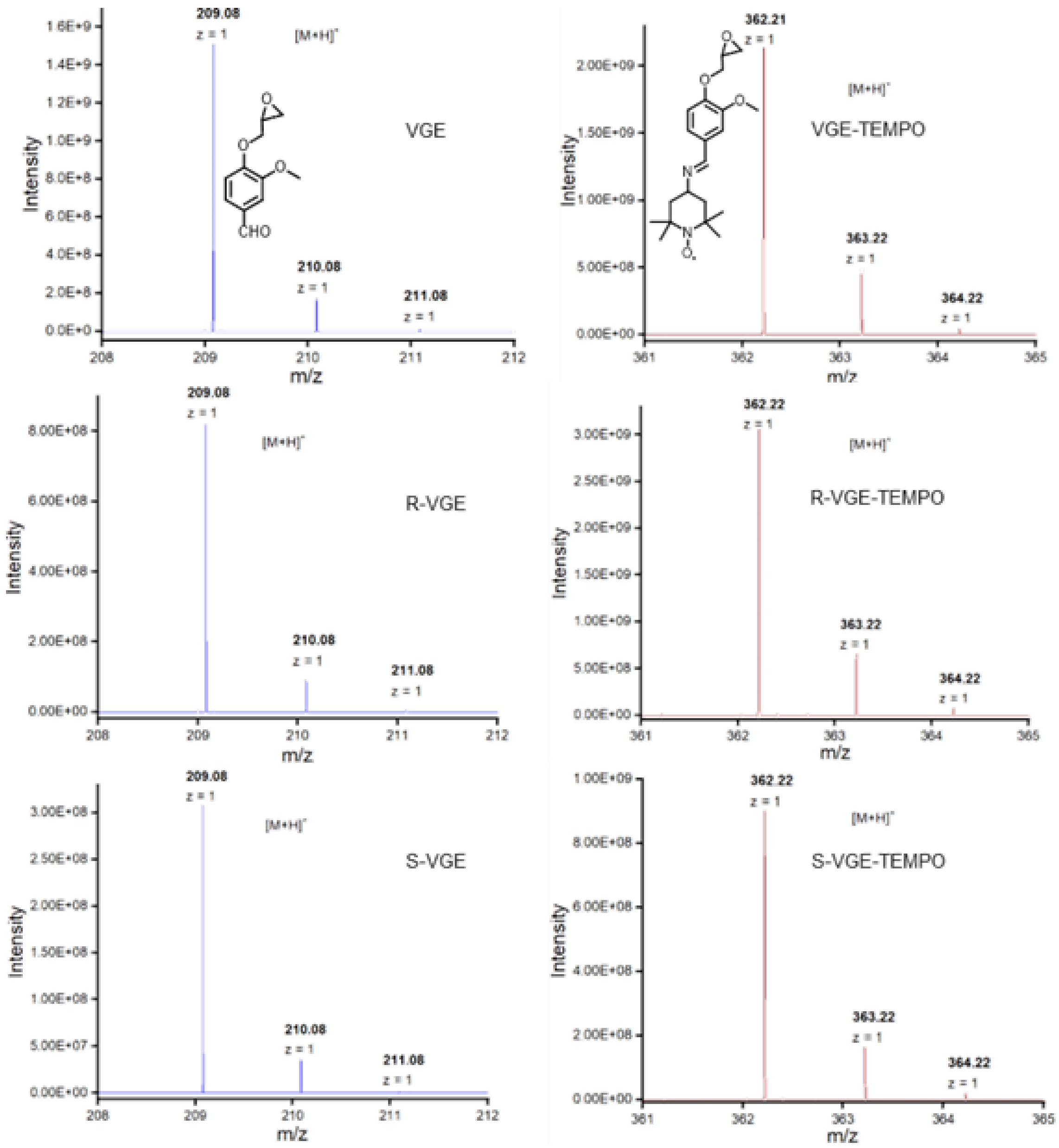

Figure S2. Mass Spectra of Vanillin-Glycidyl-Ethers (left) and their Schiff base TEMPO derivatives (right).

Synthesis of poly-monothiocarbonates (PMTCs)

Into an oven (150 °C) dried stainless-steel reactor, VGE epoxide monomer (290 mg, 250 equiv.), a Cr^{III}(salen)X (X = Cl or TFA) catalyst (4 mg, 1 equiv.), and co-catalyst PPNCl or PPNTFA (3.65 mg, 1 equiv.) were taken under an argon atmosphere using the glovebox technique. Toluene (0.4 mL) and dichloromethane (0.4 mL) solvents were added to the monomer, catalyst, and co-catalyst mixture. The reaction mixture was stirred at room temperature under 10 bar (1Mpa) pressure of COS for 12 hours. FTIR and NMR spectroscopic techniques were used to monitor the reaction

proceeding. The synthesized polymer was dissolved in dichloromethane and then precipitated in methanol to purify it. The purification procedure was repeated 3-4 times to get pure poly-thiocarbonate.

A similar synthetic procedure was followed to synthesize radical poly-monothiocarbonates (**PMTC-1'**) from VGE-TEMPO monomers. At the end of the reaction, the solvent was evaporated to dryness, and the residue was then dissolved in ethyl acetate and precipitated with hexane. This procedure, in which the residue is dissolved in ethyl acetate and precipitated in hexane, is repeated 4-5 times to purify the polymer **PMTC-1'** or **R-** and **S-PMTC-1'**.

Post-synthetic modification of poly-monothiocarbonate

Poly-monothiocarbonate **PMTC** or **R-PMTC or S-PMTC** (100 mg, 3.7 mmol) was dissolved in tetrahydrofuran solvent, and 1.2 equivalents of 4-amino-2,2,6,6-tetramethylpiperidine-1-oxyl (76 mg, 3.7 mmol) was added. The reaction mixture was refluxed at 62 ˚C for 12 hours. The reaction was monitored through FTIR and NMR spectroscopic techniques. The solvent from the orange solution was evaporated to dryness and then dissolved in ethyl acetate and precipitated in hexane 4-5 times, yielding **PMTC-1**, **R-PMTC-1,** or **S-PMTC-1**.

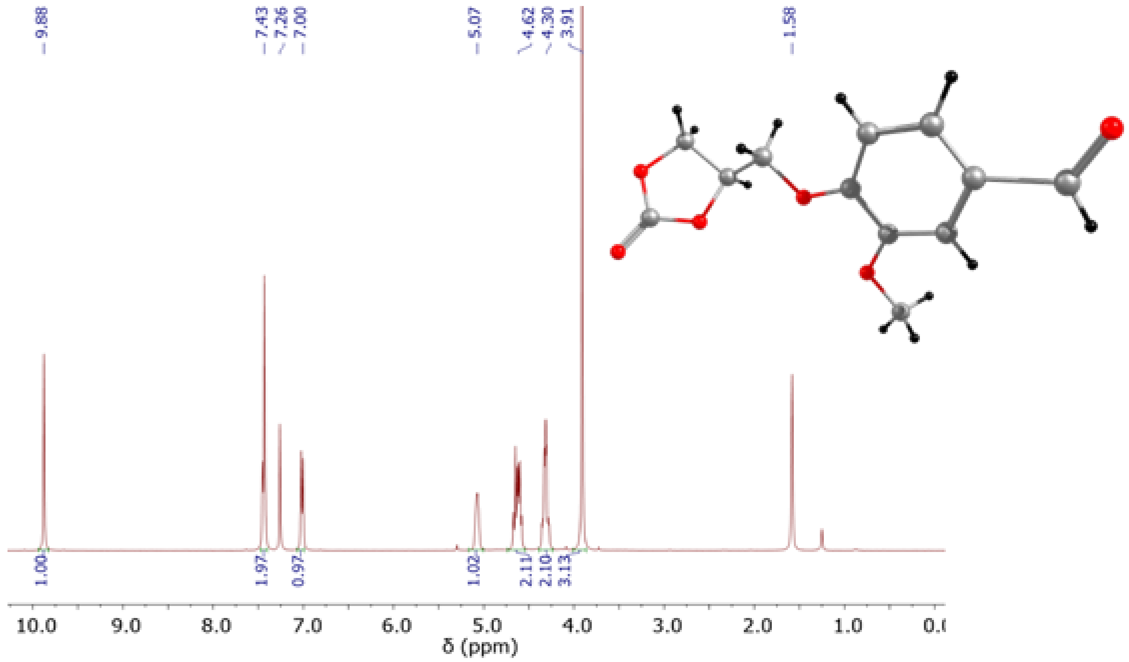

Figure S3. ^{1}H NMR spectra of cyclic carbonate recorded in $CDCl_3$ (inset X-ray structure).

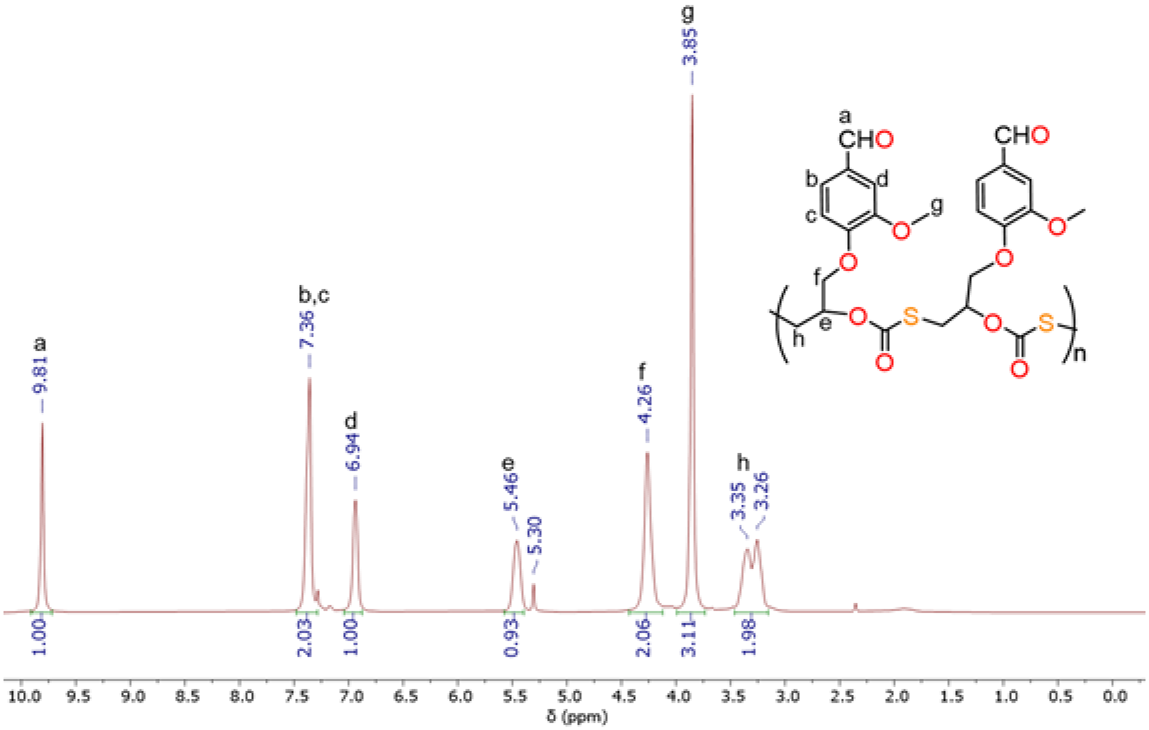

Figure S4. ^{1}H NMR spectra of poly-(monothiocarbonate) **PMTC** recorded in $CDCl_3$.

Table S1. Crystal data and structure refinement for VGE cyclic carbonate.

Identification code	P43_Cyclic	μ/mm^{-1}	1.043
Empirical formula	$C_{12}H_{11}O_6$	F(000)	524.0
Formula weight	251.21	Crystal size/mm^3	0.113 × 0.018 × 0.015
Temperature/K	110.0	Radiation	CuKα (λ = 1.54178)
Crystal system	monoclinic	2Θ range for data collection/°	8.824 to 158.232
Space group	$P2_1/n$	Index ranges	$-6 \le h \le 6$, $-13 \le k \le 12$, $-25 \le l \le 21$
a/Å	5.3776(4)	Reflections collected	11952
b/Å	10.3555(7)	Independent reflections	2209 [R_{int} = 0.0665, R_{sigma} = 0.0539]
c/Å	20.0422(13)	Data/restraints/parameters	2209/0/174
α/°	90	Goodness-of-fit on F^2	1.146
β/°	90.313(3)	Final R indexes [I>=2σ (I)]	R_1 = 0.0819, wR_2 = 0.2209
γ/°	90	Final R indexes [all data]	R_1 = 0.0900, wR_2 = 0.2257
Volume/Å^3	1116.09(13)	Largest diff. peak/hole / e Å^{-3}	0.37/-0.28
Z	4	CCDC	2502211
ρ_{calc}g/cm^3	1.495		

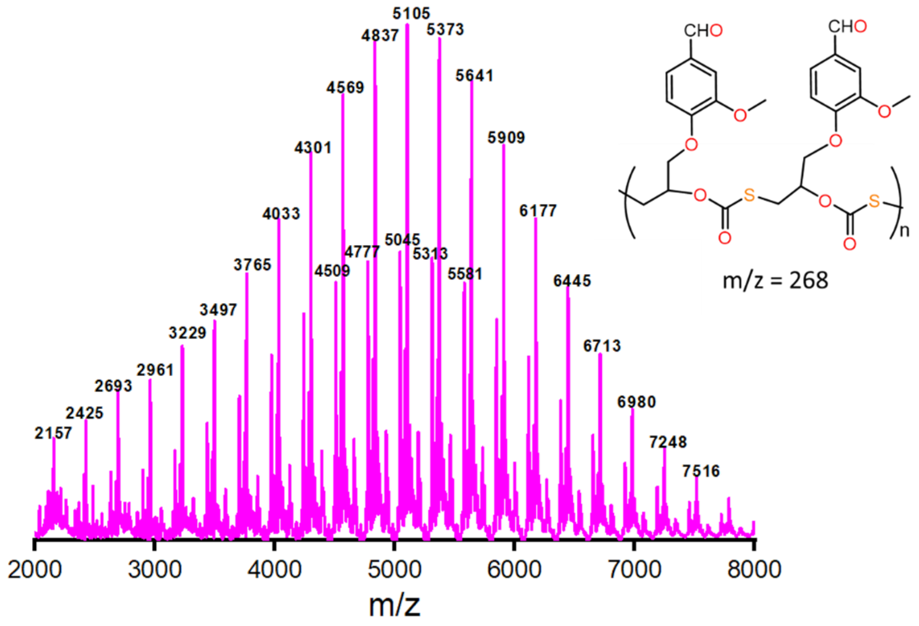

Figure S5. MALDI-TOF spectra of poly-monothiocarbonate **PMTC** recorded in THF.

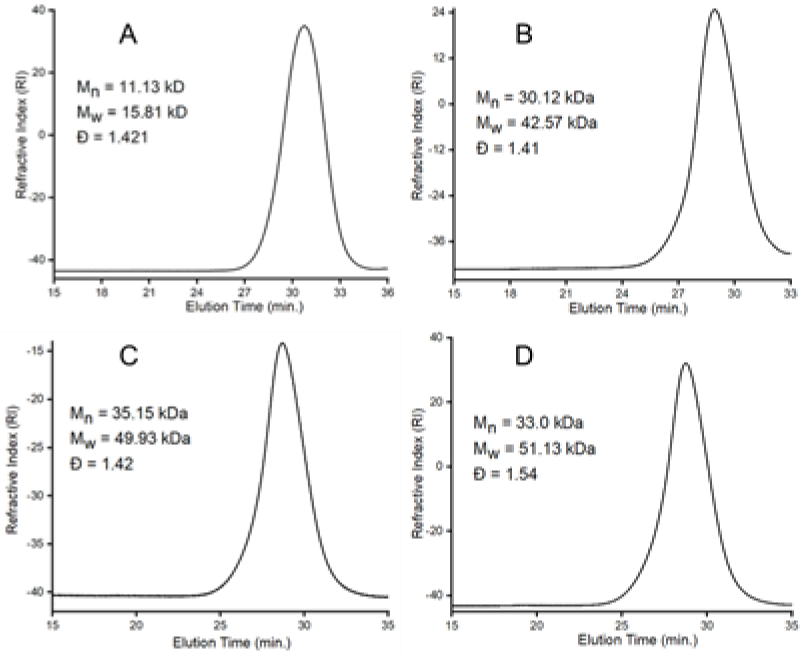

Figure S6. GPC traces for poly-monothiocarbonates **PMTCs** produced with different ratios. (A) Using Cr(salen)Cl/PPNCl catalyst and using Cr(salen)TFA/PPNTFA catalyst (B) to (D) (as shown in Table 1 entries 3-5, respectively).

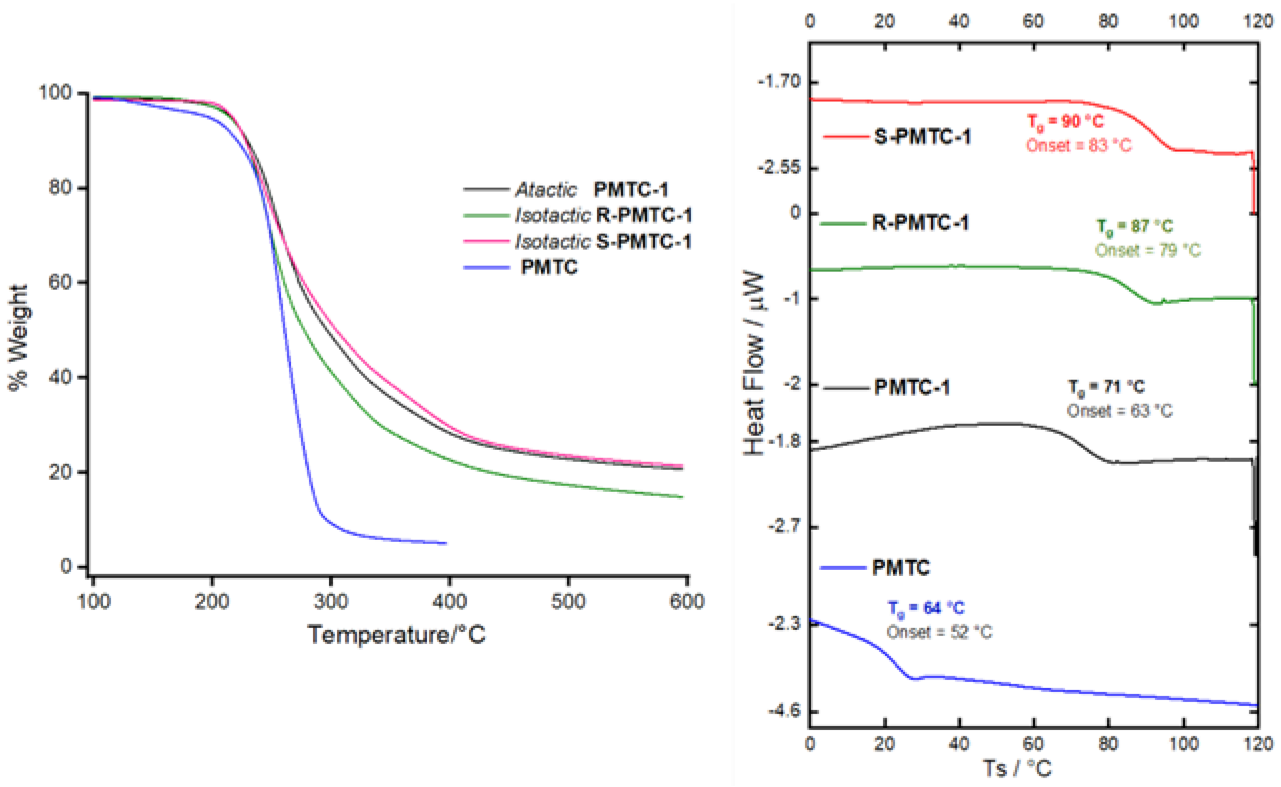

Figure S7. TGA (left) and DSC (right) data for the indicated polymers.

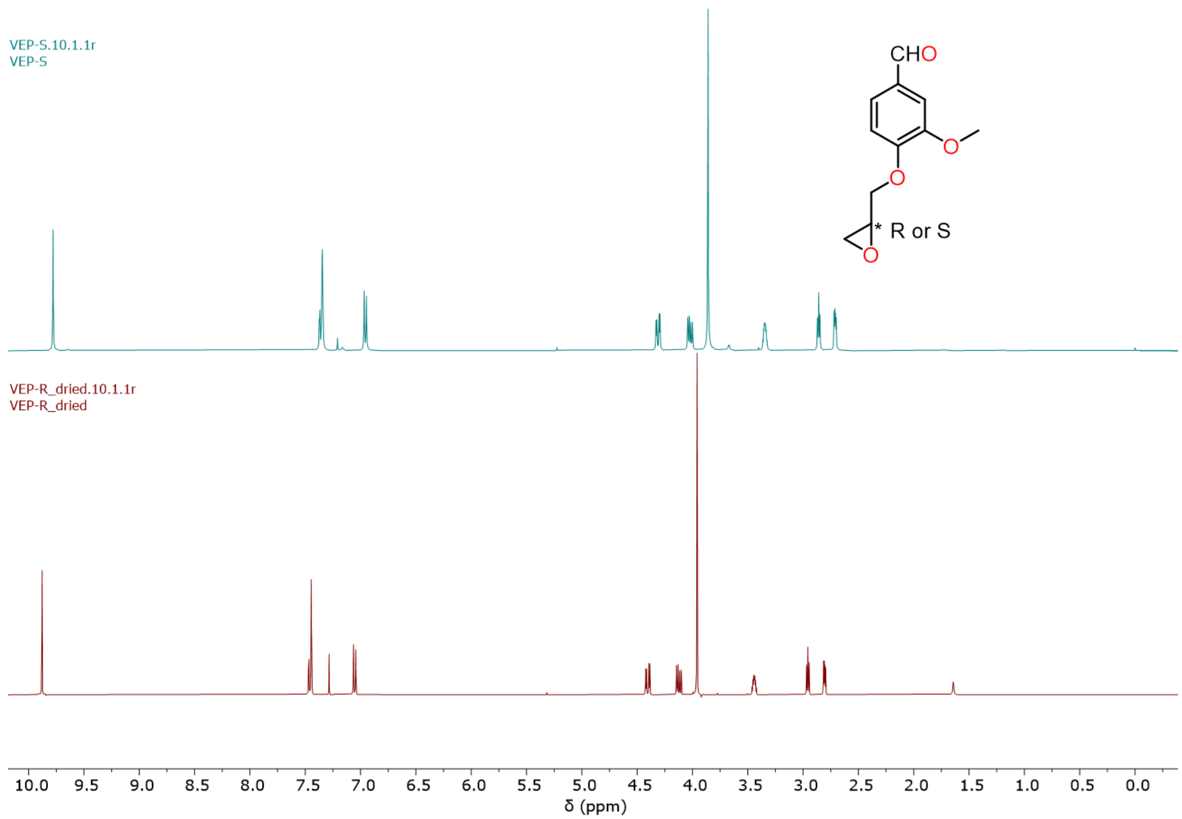

Figure S8. ^{1}H NMR spectra of R-VGE and S-VGE monomers in $CDCl_3$.

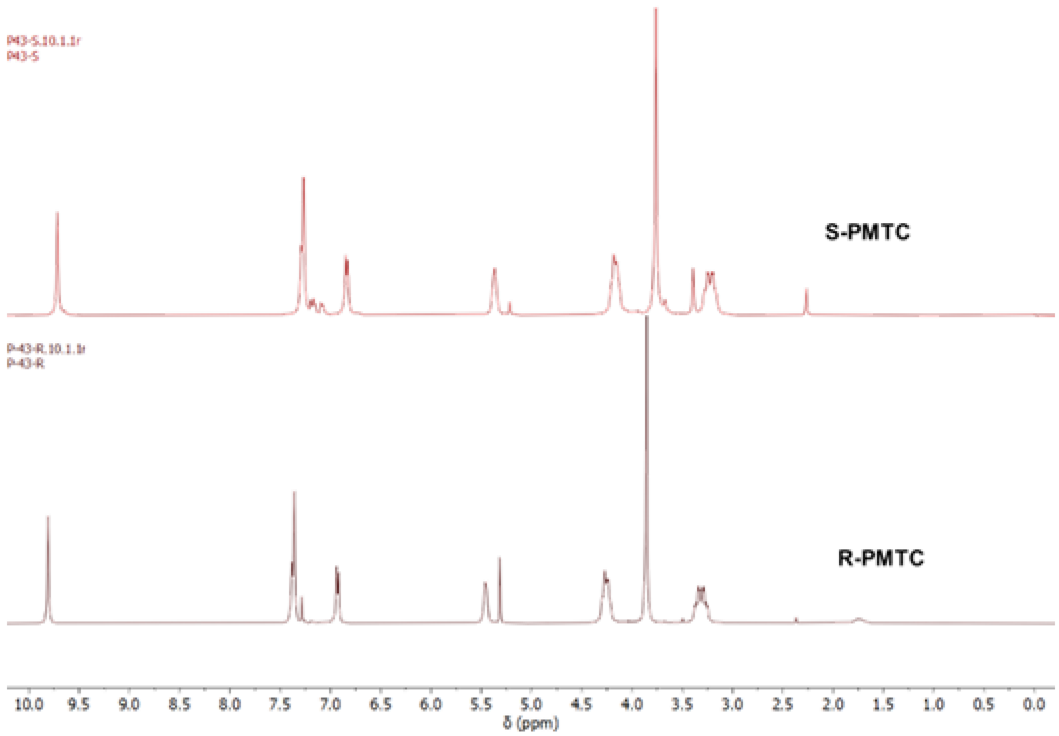

Figure S9. ^{1}H NMR spectra of isotactic **R-PMTC** and **S-PMTC** derived from R-VGE and S-VGE monomers, respectively, recorded in $CDCl_3$.

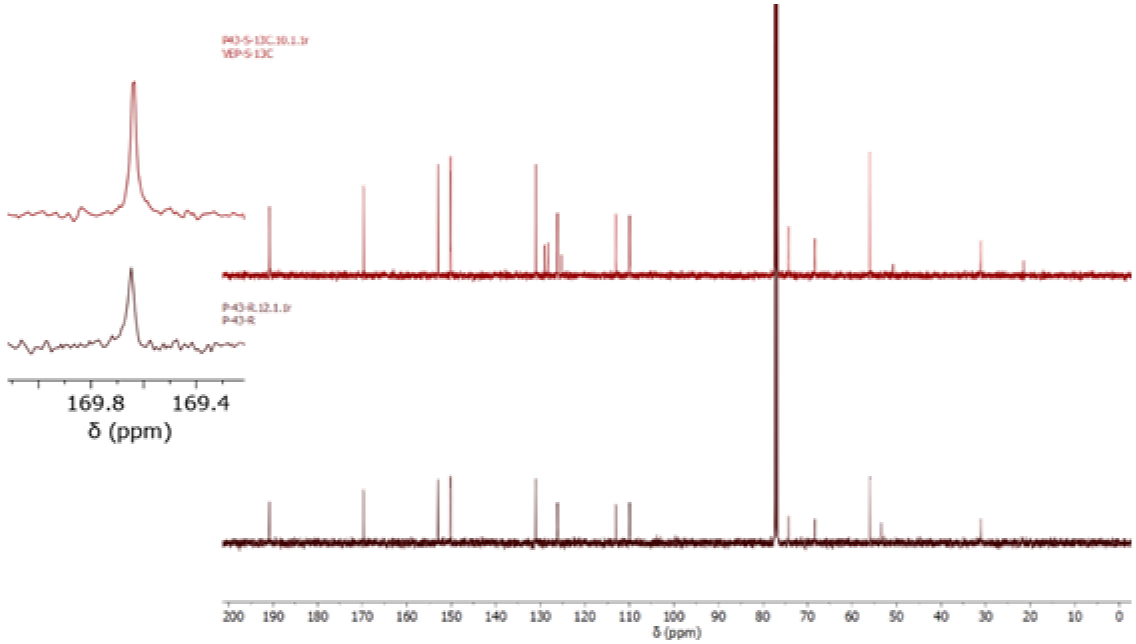

Figure S10. ^{13}C NMR spectra of poly-monothiocarbonates derived from R-VGE and S-VGE monomers in $CDCl_3$.

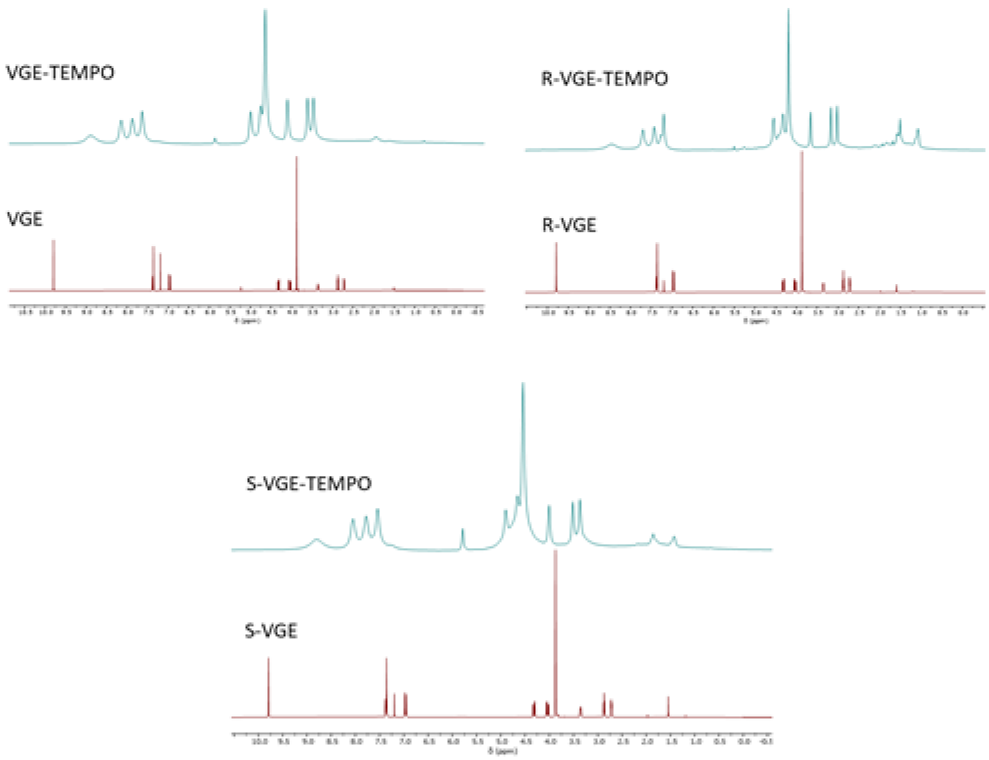

Figure S11. ^{1}H NMR spectra of VGE-TEMPO, R-VGE-TEMPO, and S-VGE-TEMPO radical monomers recorded in $CDCl_3$.

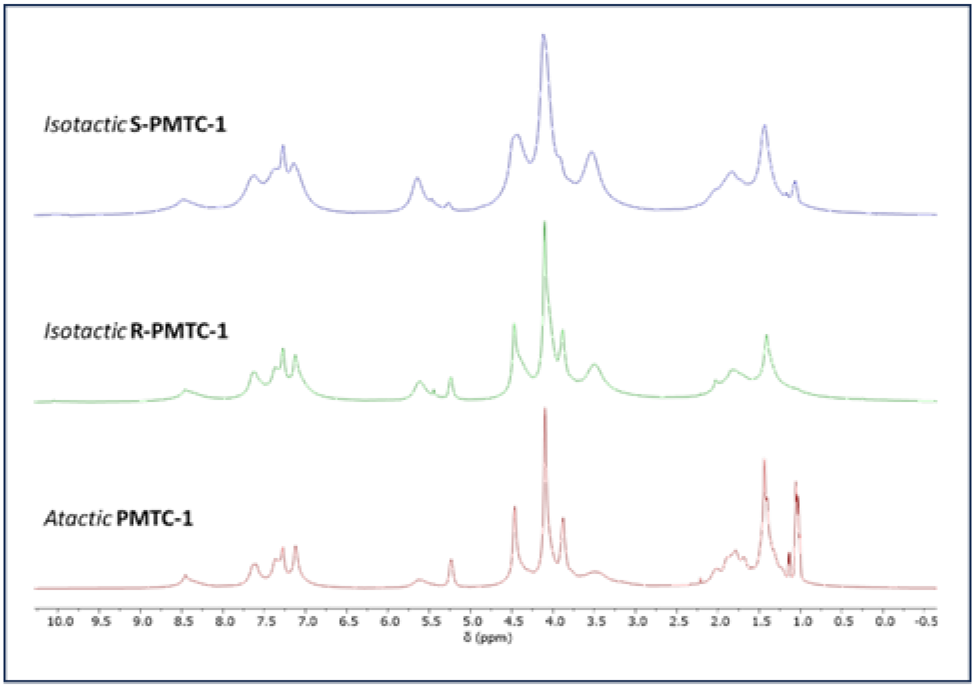

Figure S12. ^{1}H NMR spectra for *atactic* and *isotactic* radical unquenched radical polymers.

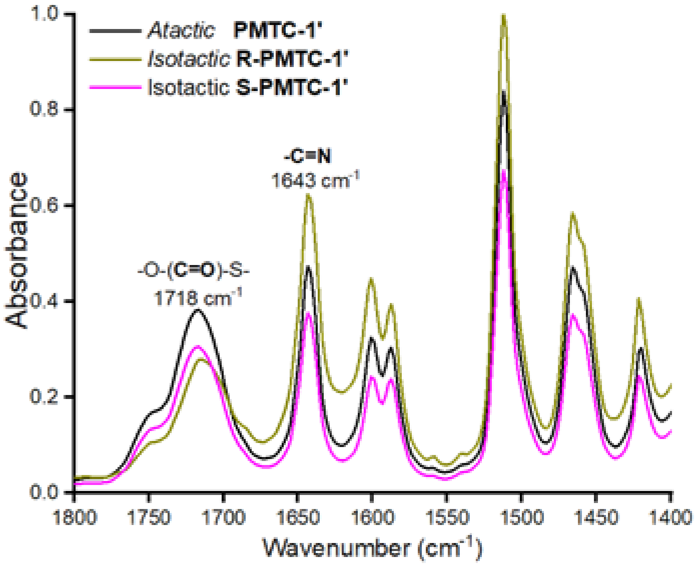

Figure S13. Infrared spectra for *atactic* and *isotactic* PMTC-1' derived from respective VGE-TEMPO monomers.

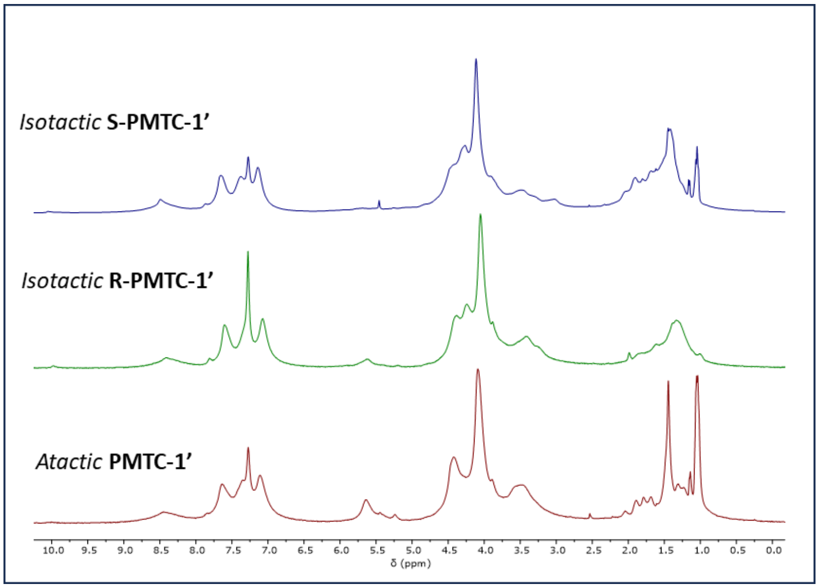

Figure S14. ^{1}H NMR spectra for *atactic* and *isotactic* PMTC-1' radical polymers.

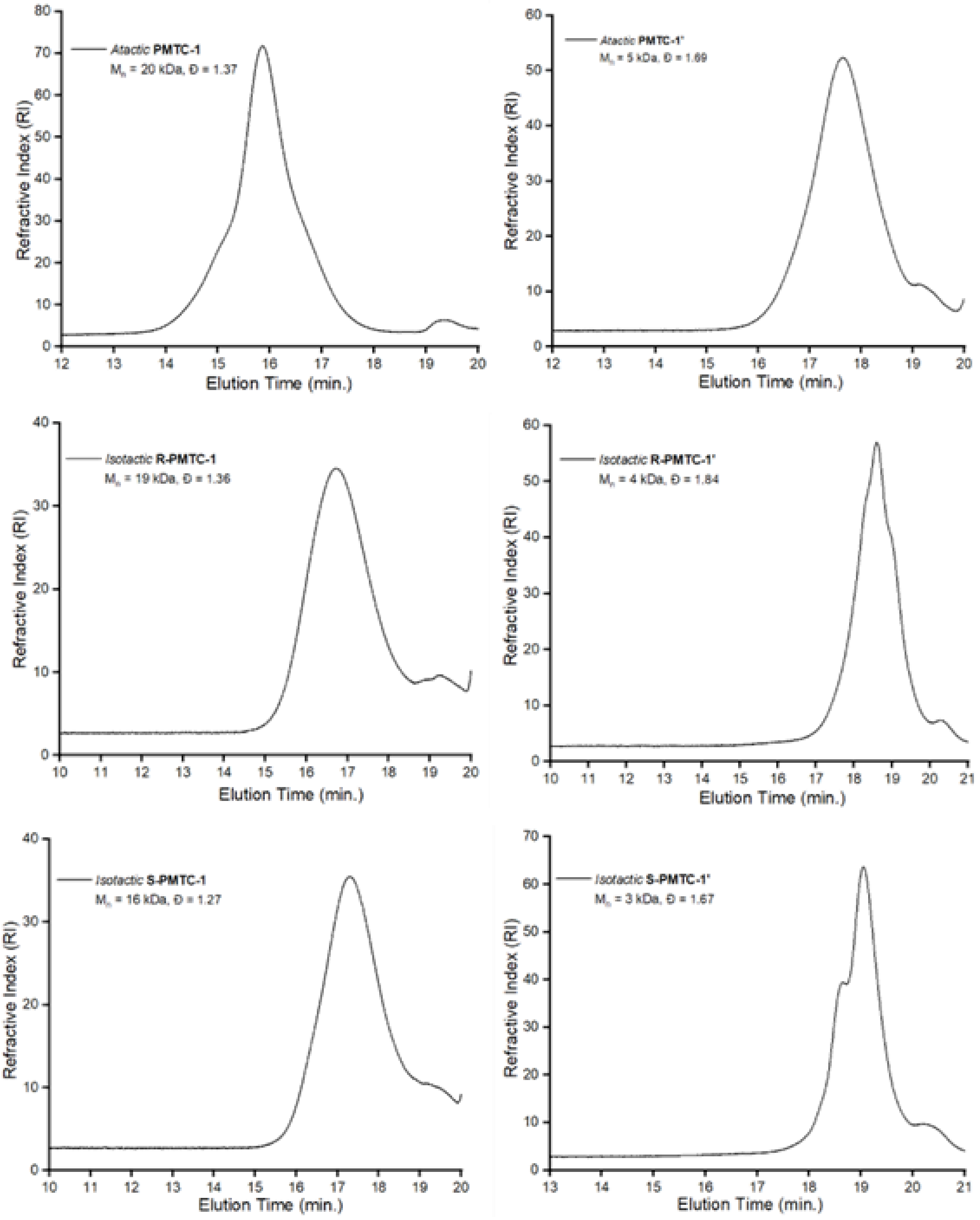

Figure S15. GPC traces of *atactic* and *isotactic* radical polymers collected in THF.

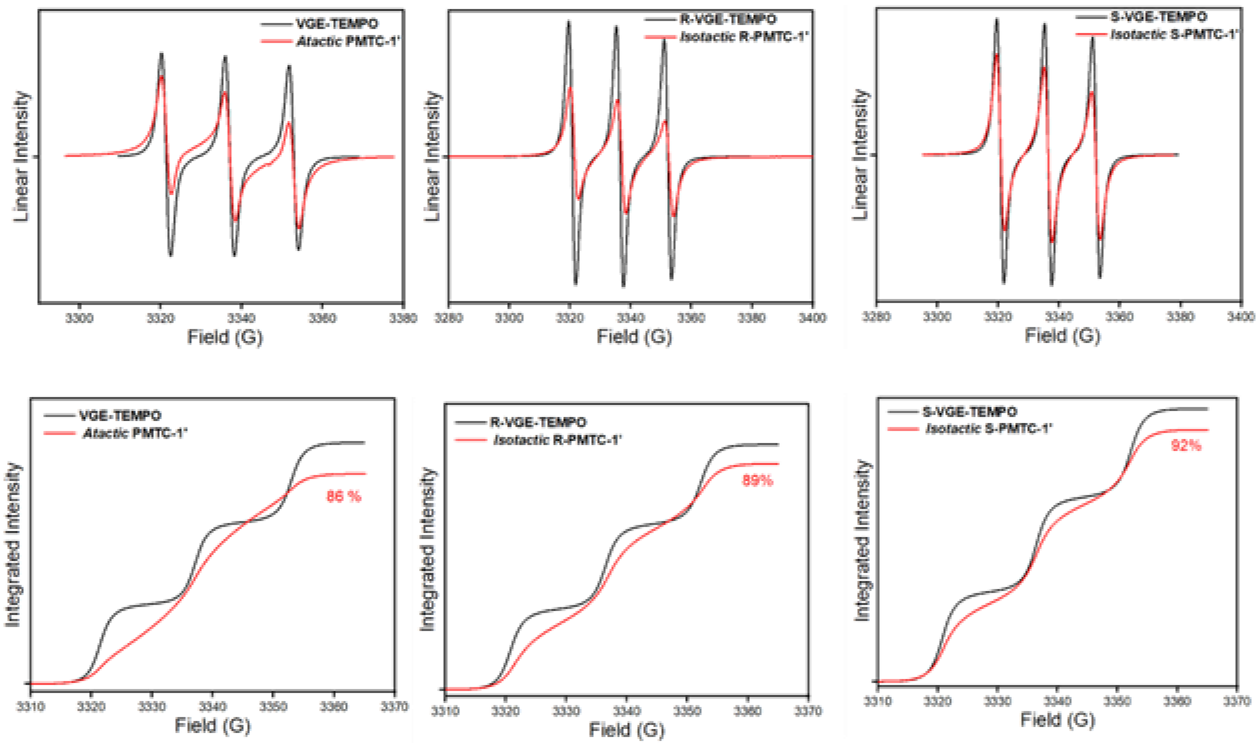

Figure S16. EPR spectra of respective VGE-TEMPOs (standard) and *atactic* **PMTC-1’**, *isotactic* **R-PMTC-1’**, and **S-PMTC-1’** were recorded for a 1 mM solution in dichloromethane. Integration suggests around >80% radical loading in these low molecular weight polymers.

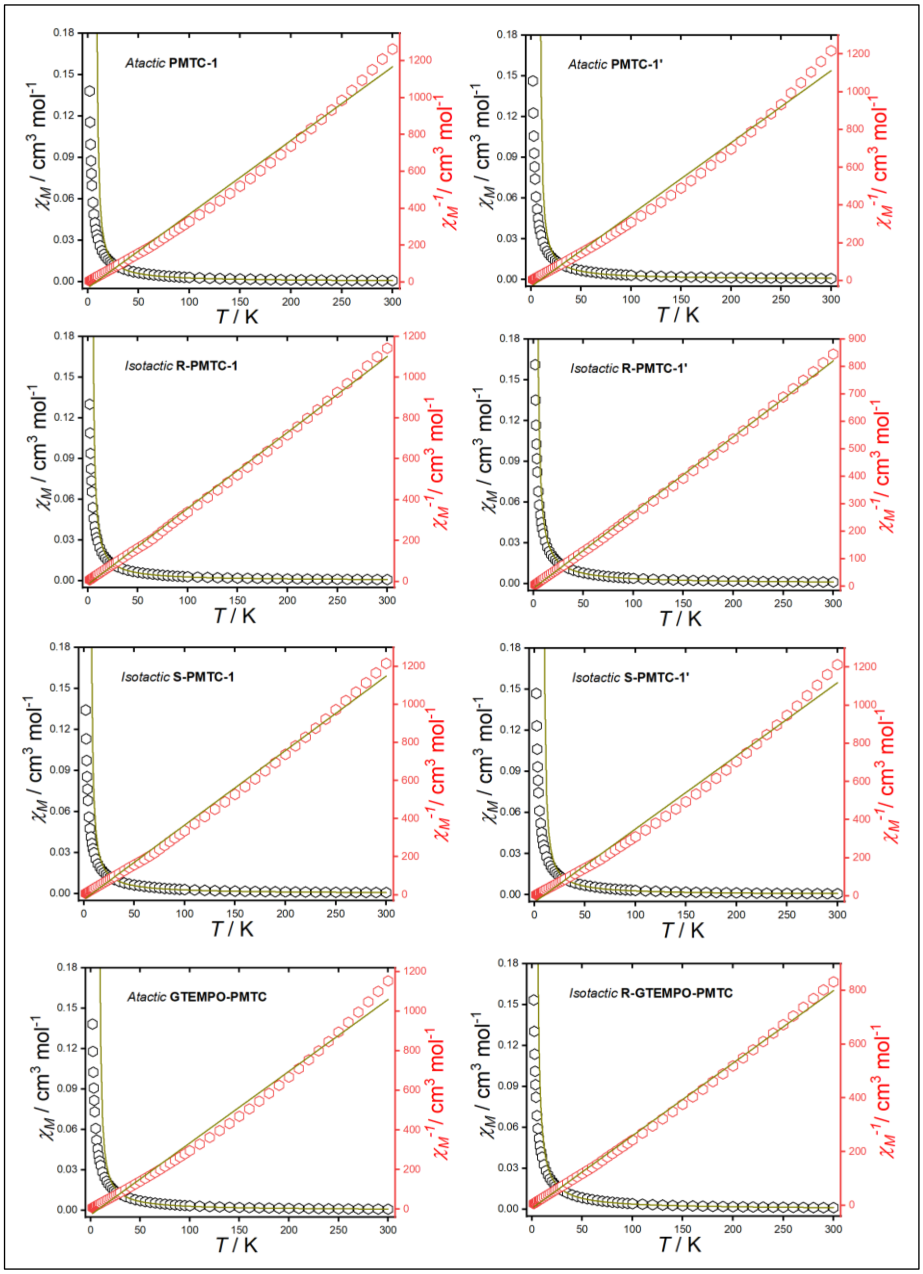

Figure S17. Temperature-dependent magnetic susceptibilities (measured under 5 kOe) plotted as $\chi_M^{-1} = f(\mathrm{T})$ and $\chi_M = f(\mathrm{T})$ for *atactic* and *isotactic* radical polymers. Solid lines represent fitted data.

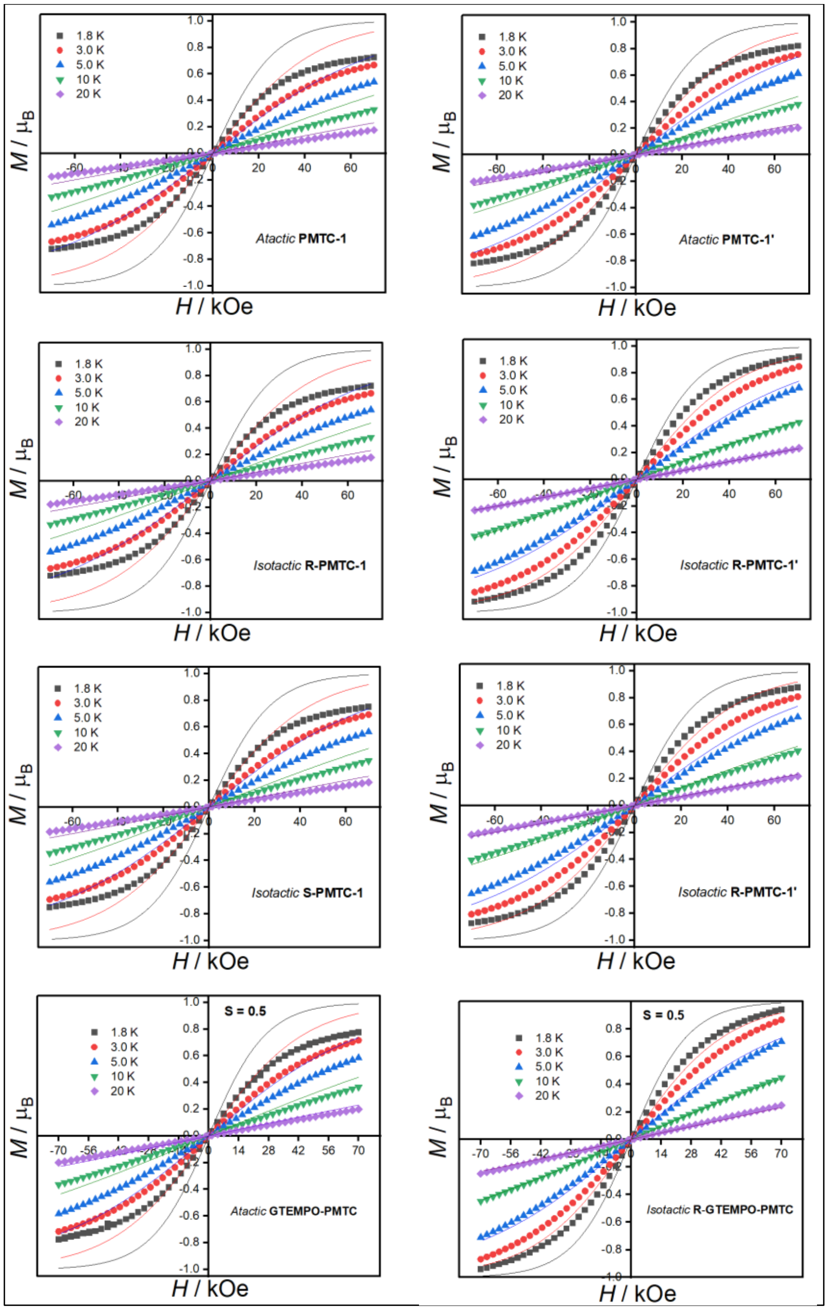

Figure S18. Field-dependent magnetization curves plotted as ($M = f$ (H)) for *atactic* and *isotactic* radical polymers. Symbols represent experimental, and the solid curves represent the fitted data.

Table S2. Measured molar magnetic susceptibility (χ_M) and magnetization saturation (M_{sat}) values for radical polymers.

**Polymers**	χ_M (cm^3 mol^{-1}) at 300 K	χ_M (cm^3 mol^{-1}) at 1.8 K	Magnetization Saturation (M_{sat} in μ_B) at +70 kOe (1.8 K)
Atactic **PMTC-1**	7.92E-4	13.86E-2	0.72
Isotactic **R-PMTC-1**	8.75E-4	12.99E-2	0.73
Isotactic **S-PMTC-1**	8.23E-4	13.41E-2	0.75
Atactic **PMTC-1'**	9.63E-4	14.6E-2	0.82
Isotactic **R-PMTC-1'**	11.80E-4	16.1E-2	0.92
Isotactic **S-PMTC-1'**	8.25E-4	14.6E-2	0.87
Atactic **GTEMPO-PMTC**	8.67E-4	13.8E-2	0.77
Isotactic **R-GTEMPO-PMTC**	12.0E-4	15.3E-2	0.94

Table S3. Direct current magnetic susceptibility data collected in the range of 0.5 kOe to 5 kOe applied magnetic field and fitted to estimate magnetic interaction θ in Kelvin.

**Applied Magnetic Field/kOe**	**0.5**	**1.0**	**1.5**	**2.0**	**2.5**	**3.0**	**3.5**	**4.0**	**4.5**	**5.0**
Atactic **PMTC-1**	-27.2	-12.3	+6.6	-0.89	-7.5	-13.5	-18.6	-23.2	-27.0	-30.5
Isotactic **R-PMTC-1**	+25.3	+17.6	+11.4	+5.4	+0.1	-4.5	-11.7	-8.5	-14.7	-17.4
Isotactic **S-PMTC-1**	+25.9	+16.9	+9.1	+2.2	-3.9	-9.2	-13.9	-18.0	-21.7	-24.9
Atactic **PMTC-1'**	-10.5	-5.4	--	--	--	--	--	--	--	-31.1
Isotactic **R-PMTC-1'**	+24.2	+15.5	+9.3	+2.5	--	--	--	--	--	-7.3
Isotactic **S-PMTC-1'**	+10.77	--	--	--	--	--	--	--	--	-13.9
Atactic **GTEMPO-PMTC**	-13.8	-18.4	-21.3	-23.2	-24.9	-26.1	-27.1	-27.9	-28.66	-28.4
Isotactic **GTEMPO-PMTC**	+4.6	+1.6	-0.8	-2.7	-4.7	-6.4	-7.8	-8.8	-9.7	-10.5

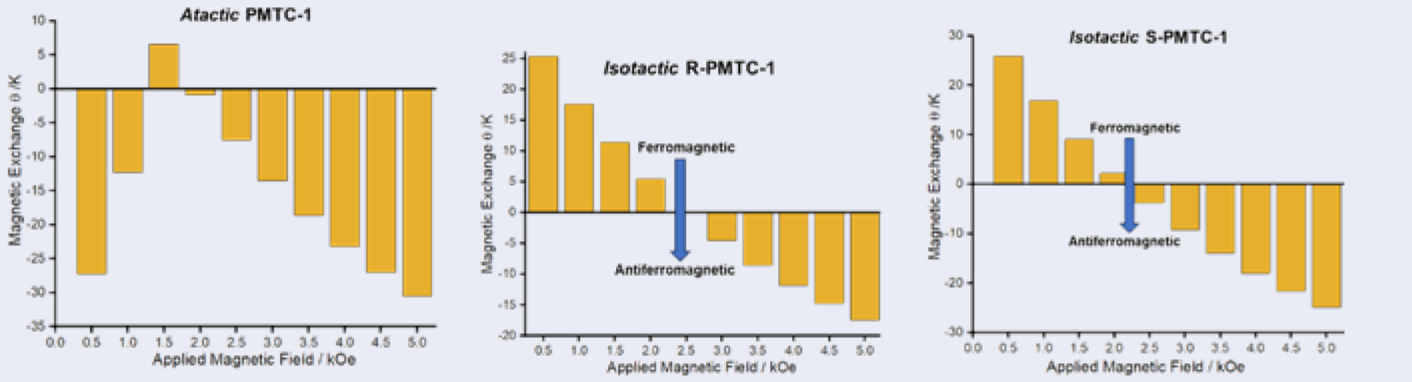

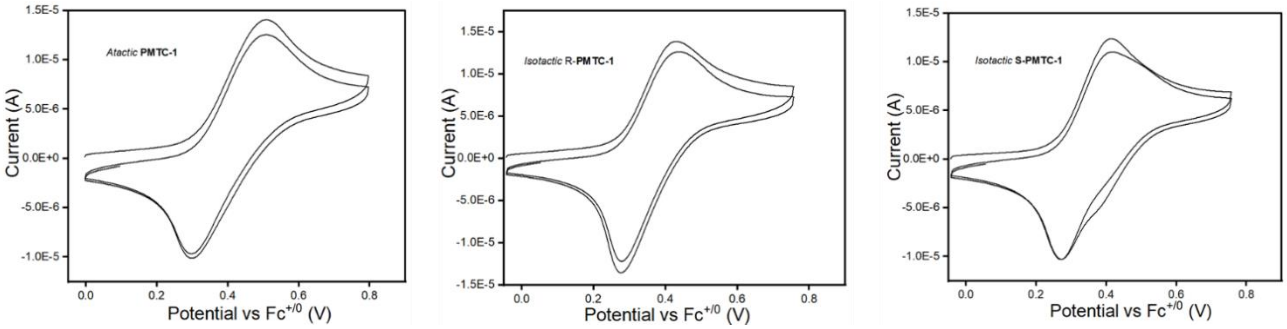

Figure S19. Cyclic voltammograms (CV) for the indicated radical polymers.

Computational Details

All calculations were performed using the Gaussian 16, revision C.01, package.[1] Spin-unrestricted density functional theory was used with D2 version of Grimme's dispersion hybrid functional UB97D[2], which incorporates a force-field-like pairwise dispersion correction, in conjunction with Petersson and coworkers' 6-311+G** basis set[3], including polarization and diffusion functions, for all atoms. Stationary points were confirmed by their zero vibrational frequencies.

We selected an oligomer containing two TEMPO units for optimization in both isotactic and atactic forms. Since isotactic and atactic polymers are built from repeating oligomeric units, this approach provides a simplified model. In the isotactic case, all side groups are oriented on the same side, whereas in the atactic case, side groups adopt a different orientation. Although this does not represent the full polymer structure, it offers insight into how two neighboring TEMPO units interact magnetically. The isotactic and atactic models were generated by modifying the stereochemistry of the carbon atoms bearing the side groups.

The oligomer (dimer) was optimized with total charges of zero and spin multiplicity of 1 and 3, respectively. DFT broken-symmetry calculations were used to optimize the singlet state. To understand the role of dispersion, we have also performed calculations on Becke's three-parameter hybrid functional B3LYP.[4] Visualization of the molecular orbitals and corresponding diagrams were performed using Chemcraft software.[5]

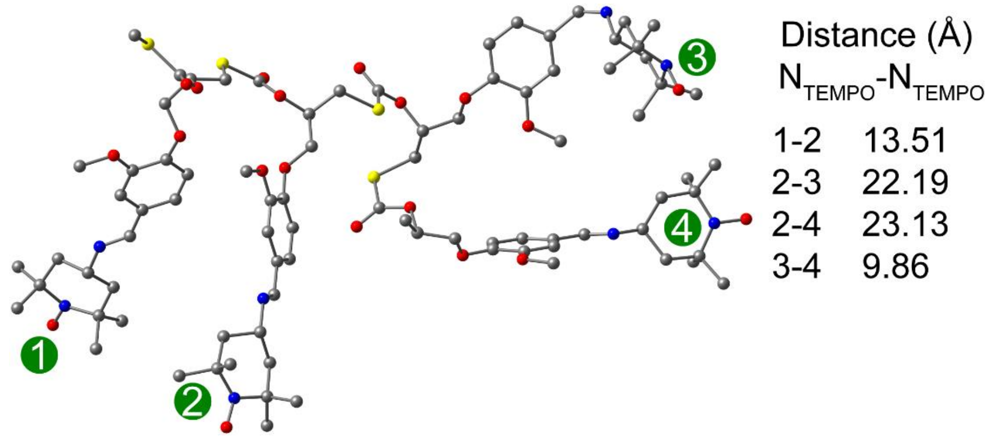

Figure S20. UB3LYP optimized geometry of oligomer containing four TEMPO units.

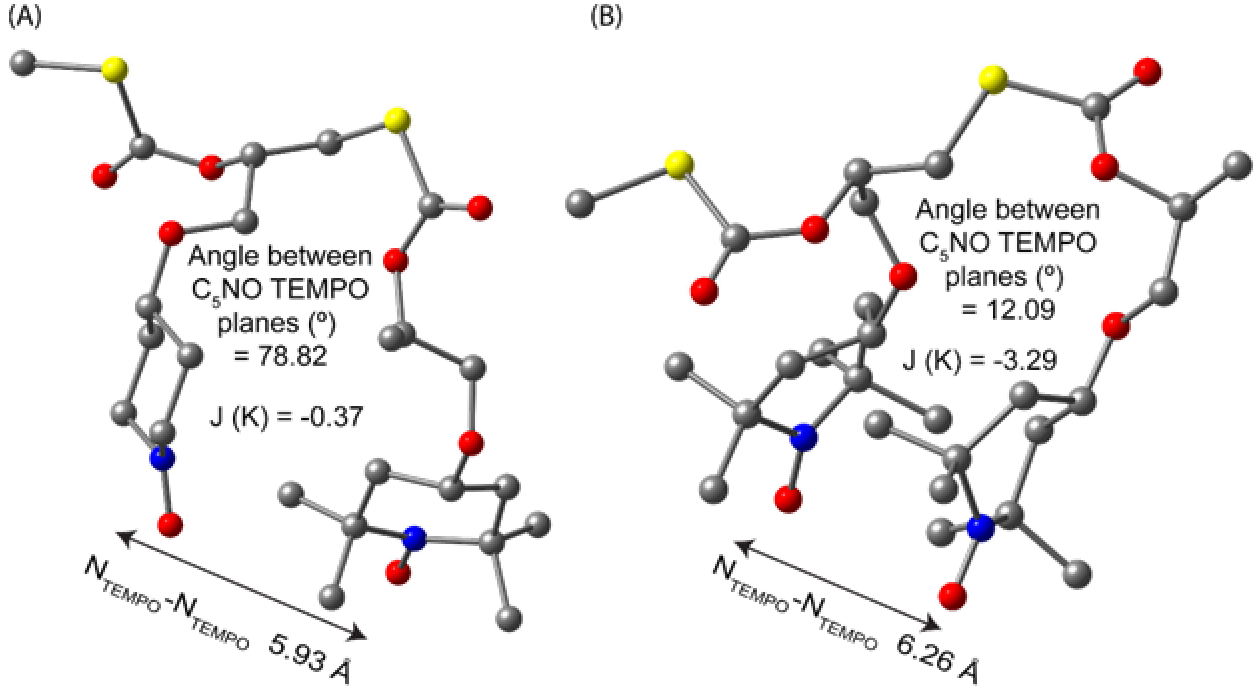

Figure S21. UB97D optimized geometry of oligomer **GTEMPO-PMTCs** containing two TEMPO units. (A) Isotactic, (B) Atactic

References:

1. Frisch, M. J. T.; Trucks, G. W.; Schlegel, H. B.;Scuseria, G. E.; Robb, M. A. C.;Cheeseman,J. R.; Scalmani, G.; Barone, V.; Mennucci, G. A. N. B.; Petersson, H.; Caricato, M.; Li, X. ; Hratchian, H. ; Izmaylov, A. F.; Bloino, J.; Zheng, G.; Sonnenberg, J. L.; Hada, M.; Ehara, M.;Toyota, K.; Fukuda, R.; Hasegawa, J.; Ishida, M.; Nakajima, Y. K. T.; Honda, O.; Nakai, H. ;Vreven, T.; Montgomery, J. A.; Peralta, Jr. J. E. O. ; Oligaro, F.; Bearpark, M.; Heyd, J. J.; Brothers, E.; Kudin, V. N.; Staroverov, K. K. N. R.; Normand, J.;Raghavachari, K.; Rendell, A. B.; Iyengar, S. S.; Tomasi, J.; Cossi, M.; Rega, J. M.; Millam, M.; Knox, J. E.; Cross, J. B.; Bakken, V.; Adamo, C. J.; Gomperts, J.; Stratmann, R. E.; Yazyev, O.; Austin, A. J.; Cammi, R.;Pomelli, J. W.; Ochterski, J. W.; Martin, R. L.; Morokuma, K. Z.; Zarkrzewski, V. G.; Voth, G. A.; Salvador, P.; Dannenberg, J. J. D.; Dapprich, S.; Daniels, A. D.; Farkas, ö.; Foresman, J. B.; Ortiz, J. V.; Cioslowski, J.; Fox, D. J. Gaussian 09, Revision B.01, Gaussian, Inc., Wallingford, CT, 2019.
2. 6. S. Grimme, "Semiempirical GGA-type density functional constructed with a long-range dispersion correction," J. Comp. Chem., 27 (2006) 1787-99
3. G. A. Petersson, A. Bennett, T. G. Tensfeldt, M. A. Al-Laham, W. A. Shirley, and J. Mantzaris, "A complete basis set model chemistry. I. The total energies of closed-shell atoms and hydrides of the first-row atoms," J. Chem. Phys., 89 (1988) 2193-218. DOI: 10.1063/1.455064.
4. A. D. Becke, J. Chem. Phys. 1993, 98, 5648; C. Lee, W. Yang, R. G. Parr, Phys. Rev. B 1988, 37, 785.
5. http://www.chemcraftprog.com.

Cartesian Coordinates of optimized geometries:

Isotactic dimer R-PMTC-1^{mod} (UB97D/6-311+G) Charge: 0, Multiplicity: 1**

6	-4.801390000	-1.392639000	-1.847439000
1	-5.169115000	-1.907788000	-2.747831000
8	-4.342846000	-2.389069000	-0.874102000
6	-3.547641000	-3.443944000	-1.167696000
8	-3.085229000	-4.118775000	-0.278184000
16	-3.290971000	-3.810278000	-2.925667000
6	-2.152508000	-5.233236000	-2.730271000
1	-2.648990000	-6.039471000	-2.182016000
1	-1.899995000	-5.556728000	-3.746859000
1	-1.253459000	-4.911960000	-2.194885000
6	-5.958189000	-0.719425000	-1.091818000
1	-5.562040000	-0.262075000	-0.183502000
1	-6.704571000	-1.477352000	-0.830368000
16	-6.776397000	0.602913000	-2.092140000
6	-6.891855000	2.016812000	-0.975436000
8	-7.245868000	3.095719000	-1.395587000
8	-6.588051000	1.707252000	0.298532000
6	-6.545229000	2.805781000	1.278972000
1	-6.252369000	3.719859000	0.748977000
6	-5.467871000	2.418106000	2.281015000
1	-5.481350000	3.137230000	3.116834000
1	-5.646447000	1.408002000	2.661685000
8	-4.196177000	2.515272000	1.610198000
6	-7.920141000	2.963202000	1.922096000
1	-7.896259000	3.781851000	2.654799000
1	-8.664587000	3.203935000	1.154655000
1	-8.216155000	2.037475000	2.434392000
6	-3.134975000	1.721527000	1.962702000
6	-1.900648000	2.373751000	2.087404000
6	-3.187611000	0.300287000	2.067043000
6	-0.716742000	1.657409000	2.257806000
1	-1.895034000	3.458455000	2.005492000
6	-1.991977000	-0.410994000	2.202566000
6	-0.753267000	0.253553000	2.265477000
1	0.238203000	2.177871000	2.309237000
1	-1.995936000	-1.495823000	2.202467000
8	-4.414588000	-0.296640000	1.985348000
6	-4.491857000	-1.704908000	2.282367000
1	-3.936031000	-2.295323000	1.545710000
1	-4.110261000	-1.897891000	3.297166000
1	-5.556316000	-1.953018000	2.224354000
6	0.513466000	-0.482210000	2.210478000
1	1.367510000	0.026799000	2.679422000
7	0.634891000	-1.602585000	1.590136000
6	1.938231000	-2.286650000	1.536781000
6	3.686092000	-3.200354000	-0.118859000
6	4.359172000	-2.548036000	2.357357000
7	4.731386000	-2.858022000	0.922037000
6	4.288087000	-2.903631000	-1.504188000
1	5.168409000	-3.529797000	-1.682689000
1	3.528902000	-3.114794000	-2.269324000
1	4.579573000	-1.849159000	-1.579673000
6	3.339161000	-4.703250000	-0.030589000
1	2.722572000	-4.980395000	-0.897080000
1	4.264673000	-5.291755000	-0.050083000
1	2.779400000	-4.949259000	0.878256000
6	5.503484000	-1.724009000	2.972395000
1	5.240224000	-1.476312000	4.009722000
1	6.438649000	-2.293426000	2.961866000
1	5.648388000	-0.793128000	2.411521000
6	4.208056000	-3.869096000	3.145422000
1	5.100477000	-4.487389000	2.989383000
1	4.115810000	-3.641099000	4.216538000
1	3.323107000	-4.437269000	2.839368000
6	2.446249000	-2.309615000	0.084408000
1	2.716220000	-1.281184000	-0.193273000
1	1.656818000	-2.653892000	-0.597324000
6	3.067077000	-1.707297000	2.398941000
1	2.757825000	-1.622550000	3.450979000
1	3.298614000	-0.694775000	2.035959000
1	1.730933000	-3.320178000	1.852043000
8	5.912930000	-3.312793000	0.722473000
6	-3.667113000	-0.457324000	-2.260570000
1	-2.796233000	-1.052475000	-2.559133000
1	-4.005524000	0.141521000	-3.121750000
8	-3.339770000	0.425265000	-1.174987000
6	-2.036515000	0.871469000	-1.168062000
6	-0.969261000	-0.012697000	-0.865861000
6	-1.777925000	2.229533000	-1.390320000
6	0.325347000	0.498036000	-0.825425000
6	-0.473296000	2.732698000	-1.310758000
1	-2.624283000	2.881351000	-1.598248000
6	0.592135000	1.862782000	-1.014419000
1	1.147390000	-0.165143000	-0.599996000
1	-0.285680000	3.795349000	-1.464947000
8	-1.231440000	-1.326038000	-0.602170000
1	-0.591222000	-1.630792000	0.094016000
6	1.971796000	2.357919000	-0.873262000
7	2.893525000	1.631205000	-0.354519000
6	4.282879000	2.084615000	-0.277296000
6	5.150695000	0.990063000	-0.921854000
6	4.637428000	3.421951000	-0.938874000
6	6.667002000	1.219634000	-0.783354000
1	4.883929000	0.938419000	-1.988671000
6	6.120247000	3.813343000	-0.775730000
1	4.400763000	3.350615000	-2.012077000
7	7.017498000	2.649259000	-1.134176000
1	2.144201000	3.385106000	-1.237801000
1	4.911461000	0.017072000	-0.473892000
1	4.035209000	4.243963000	-0.525104000
1	4.527848000	2.131363000	0.796389000
6	7.404322000	0.297867000	-1.771270000
1	8.487872000	0.424462000	-1.676703000
1	7.138669000	-0.743646000	-1.546426000
1	7.108418000	0.530562000	-2.803064000
6	7.151858000	0.913928000	0.651437000
1	7.089305000	-0.167460000	0.827918000
1	8.195688000	1.233514000	0.757434000
1	6.544291000	1.425654000	1.407769000
6	6.427806000	4.262843000	0.671145000
1	7.506518000	4.431696000	0.774710000
1	5.896809000	5.202223000	0.880471000
1	6.113221000	3.516214000	1.409052000
6	6.443107000	4.965360000	-1.744297000
1	5.777230000	5.811097000	-1.523849000
1	7.484637000	5.283084000	-1.629951000
1	6.282455000	4.646282000	-2.782692000

8	8.264651000	2.914444000	-1.264634000

Isotactic dimer R-PMTC-1^{mod} (UB97D/6-311+G**) Charge: 0, Multiplicity: 3

6	4.853281000	1.326673000	-1.735098000
1	5.244995000	1.842664000	-2.624837000
8	4.385920000	2.322958000	-0.765746000
6	3.606711000	3.386944000	-1.069097000
8	3.133262000	4.061474000	-0.185170000
16	3.388361000	3.765236000	-2.829695000
6	2.259554000	5.197776000	-2.648750000
1	2.753469000	5.997486000	-2.088736000
1	2.027818000	5.527354000	-3.668327000
1	1.348450000	4.882573000	-2.130397000
6	5.986950000	0.636756000	-0.959631000
1	5.567371000	0.180136000	-0.061474000
1	6.736747000	1.384740000	-0.679937000
16	6.810078000	-0.690654000	-1.949121000
6	6.875708000	-2.114747000	-0.841298000
8	7.223464000	-3.195712000	-1.261440000
8	6.545581000	-1.810496000	0.427314000
6	6.467150000	-2.915468000	1.398279000
1	6.170056000	-3.820822000	0.855801000
6	5.377413000	-2.518446000	2.383089000
1	5.365491000	-3.242833000	3.214358000
1	5.563574000	-1.513398000	2.773394000
8	4.116566000	-2.593257000	1.689260000
6	7.827095000	-3.099368000	2.065831000
1	7.776755000	-3.923384000	2.791130000
1	8.582101000	-3.345500000	1.310533000
1	8.127535000	-2.182472000	2.591273000
6	3.061754000	-1.784097000	2.025419000
6	1.814974000	-2.416971000	2.123488000
6	3.135047000	-0.364412000	2.137773000
6	0.639572000	-1.682585000	2.274887000
1	1.793619000	-3.501044000	2.036145000
6	1.948501000	0.365321000	2.253768000
6	0.698370000	-0.279514000	2.289646000
1	-0.324369000	-2.187908000	2.305050000
1	1.969660000	1.449947000	2.258105000
8	4.372691000	0.213245000	2.082227000
6	4.466917000	1.618322000	2.389288000
1	3.935288000	2.221960000	1.645532000
1	4.068643000	1.811665000	3.397589000
1	5.536206000	1.849334000	2.353616000
6	-0.554871000	0.476940000	2.209916000
1	-1.426870000	-0.017634000	2.660903000
7	-0.644443000	1.598827000	1.586884000
6	-1.934701000	2.304312000	1.504167000
6	-3.632956000	3.237591000	-0.192176000
6	-4.367586000	2.609160000	2.272703000
7	-4.704934000	2.917751000	0.828506000
6	-4.210813000	2.942489000	-1.588082000
1	-5.076974000	3.581695000	-1.788317000
1	-3.432617000	3.136891000	-2.338420000
1	-4.517737000	1.892435000	-1.663623000
6	-3.264157000	4.735294000	-0.104532000
1	-2.625414000	4.997930000	-0.959414000
1	-4.179616000	5.338178000	-0.146319000
1	-2.719612000	4.977354000	0.814551000
6	-5.537854000	1.807328000	2.867911000
1	-5.300826000	1.561760000	3.912041000
1	-6.463219000	2.391641000	2.833971000
1	-5.685654000	0.875360000	2.309518000
6	-4.210918000	3.931666000	3.057276000
1	-5.089879000	4.563433000	2.880204000
1	-4.143957000	3.707792000	4.131136000
1	-3.310910000	4.483967000	2.766423000
6	-2.411623000	2.328824000	0.041307000
1	-2.691094000	1.303320000	-0.237863000
1	-1.602762000	2.657886000	-0.624982000
6	-3.090750000	1.747460000	2.344911000
1	-2.804816000	1.662096000	3.403480000
1	-3.331801000	0.737363000	1.981340000
1	-1.716808000	3.335629000	1.819468000
8	-5.874343000	3.391106000	0.602408000
6	3.717908000	0.405743000	-2.176818000
1	2.859176000	1.011457000	-2.488942000
1	4.067540000	-0.191119000	-3.034857000
8	3.360082000	-0.480593000	-1.103939000
6	2.051445000	-0.910209000	-1.122898000
6	0.989886000	-0.013830000	-0.836778000
6	1.780063000	-2.263935000	-1.356088000
6	-0.311489000	-0.508410000	-0.822731000
6	0.467889000	-2.750828000	-1.303725000
1	2.621978000	-2.925453000	-1.550992000
6	-0.591873000	-1.868736000	-1.023592000
1	-1.129380000	0.163922000	-0.609752000
1	0.269816000	-3.810260000	-1.466732000
8	1.263814000	1.294994000	-0.563229000
1	0.613151000	1.606937000	0.120169000
6	-1.980405000	-2.346326000	-0.912177000
7	-2.902523000	-1.609704000	-0.408331000
6	-4.299192000	-2.044183000	-0.361066000
6	-5.139238000	-0.932149000	-1.012367000
6	-4.659849000	-3.370817000	-1.040723000
6	-6.660904000	-1.141216000	-0.903914000
1	-4.851852000	-0.875540000	-2.073564000
6	-6.150868000	-3.742420000	-0.908830000
1	-4.401756000	-3.293841000	-2.108584000
7	-7.024883000	-2.562713000	-1.273615000
1	-2.159161000	-3.369339000	-1.285329000
1	-4.894665000	0.033716000	-0.551968000
1	-4.077386000	-4.204845000	-0.622771000
1	-4.565180000	-2.096463000	0.707342000
6	-7.366794000	-0.200691000	-1.897088000
1	-8.453584000	-0.312658000	-1.823504000
1	-7.090637000	0.835003000	-1.658449000
1	-7.055237000	-0.428928000	-2.925264000
6	-7.167775000	-0.840941000	0.524433000
1	-7.094671000	0.238130000	0.710761000
1	-8.217403000	-1.147659000	0.608946000
1	-6.580612000	-1.366525000	1.287375000
6	-6.491773000	-4.199855000	0.528062000
1	-7.574457000	-4.354567000	0.609996000
1	-5.977786000	-5.148278000	0.739069000
1	-6.180870000	-3.463961000	1.278223000
6	-6.471638000	-4.881401000	-1.893372000
1	-5.822111000	-5.738445000	-1.667876000
1	-7.519555000	-5.185091000	-1.801412000
1	-6.286825000	-4.555820000	-2.925714000
8	-8.273021000	-2.809052000	-1.429195000

Isotactic dimer R-PMTC-1^{mod} (UB3LYP/6-311+G**) Charge: 0, Multiplicity: 3

6	-1.926332000	5.195885000	1.231188000
1	-2.647195000	5.920945000	1.627191000
8	-0.603732000	5.494348000	1.750467000
6	-0.377240000	6.078500000	2.946049000
8	0.748565000	6.258501000	3.334075000
16	-1.832459000	6.578217000	3.897250000
6	-0.948596000	7.311985000	5.316413000
1	-0.349497000	8.161195000	4.989300000
1	-1.720596000	7.639830000	6.014700000
1	-0.307096000	6.565230000	5.783024000
6	-1.766870000	5.373709000	-0.281319000
1	-1.108726000	4.599484000	-0.671573000
1	-1.332552000	6.354604000	-0.481323000
16	-3.397085000	5.244901000	-1.130242000
6	-2.969919000	4.656488000	-2.772422000
8	-3.827318000	4.437466000	-3.593838000
8	-1.646761000	4.520920000	-2.947069000
6	-1.194617000	4.031395000	-4.247254000
1	-1.921893000	3.295168000	-4.598569000
6	0.140052000	3.357586000	-3.989467000
1	0.640017000	3.167441000	-4.949394000
1	0.777641000	4.008491000	-3.380945000
8	-0.099619000	2.115851000	-3.318315000
6	-1.091729000	5.198072000	-5.221812000
1	-0.765285000	4.843376000	-6.204185000
1	-2.067877000	5.672689000	-5.338920000
1	-0.375314000	5.944573000	-4.865175000
6	1.032691000	1.441643000	-2.911502000
6	1.435188000	0.307702000	-3.606406000
6	1.736963000	1.855001000	-1.753085000
6	2.538526000	-0.431285000	-3.175918000
1	0.862703000	0.012346000	-4.479890000
6	2.839370000	1.116559000	-1.332021000
6	3.246712000	-0.027936000	-2.040574000
1	2.846955000	-1.318090000	-3.722531000
1	3.406698000	1.392703000	-0.452473000
8	1.259764000	2.966716000	-1.129855000
6	1.853939000	3.340480000	0.114991000
1	1.793041000	2.520688000	0.838358000
1	2.902093000	3.632529000	-0.020045000
1	1.277657000	4.185505000	0.487730000
6	4.413127000	-0.812271000	-1.595921000
1	4.637272000	-1.687802000	-2.220617000
7	5.099391000	-0.488434000	-0.571435000
6	6.246317000	-1.279196000	-0.133163000
6	7.197607000	-2.389857000	1.993000000
6	7.881447000	-3.241540000	-0.431470000
7	7.824157000	-3.387911000	1.061868000
6	6.724439000	-3.166600000	3.232703000
1	7.568807000	-3.648366000	3.727018000
1	6.240365000	-2.480798000	3.934808000
1	6.003555000	-3.939972000	2.951391000
6	8.263889000	-1.357641000	2.416890000
1	7.874541000	-0.726203000	3.221730000
1	9.150913000	-1.881996000	2.780071000
1	8.562689000	-0.703424000	1.593585000
6	7.935774000	-4.661623000	-1.019261000
1	7.960875000	-4.608540000	-2.112201000
1	8.823545000	-5.187228000	-0.666066000
1	7.054628000	-5.236934000	-0.719830000
6	9.171925000	-2.486077000	-0.815691000
1	10.026863000	-2.966320000	-0.334065000
1	9.316958000	-2.517929000	-1.900346000
1	9.149421000	-1.436623000	-0.510940000
6	5.989043000	-1.717819000	1.316069000
1	5.140527000	-2.413835000	1.309372000
1	5.683115000	-0.852720000	1.912503000
6	6.620016000	-2.520253000	-0.950451000
1	6.793932000	-2.264609000	-2.002376000
1	5.777198000	-3.223433000	-0.928234000
1	7.091485000	-0.576904000	-0.136551000
8	8.722969000	-4.146470000	1.580043000
6	-2.354431000	3.798632000	1.668339000
1	-2.333494000	3.724664000	2.759021000
1	-3.380796000	3.619080000	1.327165000
8	-1.458895000	2.854141000	1.078981000
6	-1.910987000	1.570548000	0.887471000
6	-2.405268000	0.781678000	1.949500000
6	-1.802497000	1.019162000	-0.387488000
6	-2.804839000	-0.526205000	1.706696000
6	-2.189000000	-0.299894000	-0.619838000
1	-1.407660000	1.638997000	-1.185481000
6	-2.700570000	-1.082161000	0.422067000
1	-3.191655000	-1.148071000	2.510235000
1	-2.098937000	-0.718090000	-1.618317000
8	-2.458872000	1.354217000	3.193208000
1	-2.818454000	0.707422000	3.814626000
6	-3.113902000	-2.471025000	0.161845000
7	-3.581315000	-3.225315000	1.078311000
6	-3.993455000	-4.599574000	0.804111000
6	-5.462992000	-4.744646000	1.226249000
6	-3.854336000	-5.120031000	-0.630434000
6	-5.987740000	-6.191632000	1.189817000
1	-6.066953000	-4.119977000	0.555815000
6	-4.261789000	-6.599463000	-0.791410000
1	-4.482888000	-4.506803000	-1.289283000
7	-5.556918000	-6.867205000	-0.080291000
1	-2.981373000	-2.796672000	-0.878728000
1	-5.595900000	-4.344010000	2.236081000
1	-2.823568000	-5.019986000	-0.990325000
1	-3.379277000	-5.218398000	1.473236000
6	-7.525244000	-6.180316000	1.194176000
1	-7.914964000	-7.198633000	1.170316000
1	-7.887751000	-5.677831000	2.096282000
1	-7.909463000	-5.644367000	0.321252000
6	-5.482439000	-7.014266000	2.394093000
1	-5.927251000	-6.635094000	3.319594000
1	-5.774741000	-8.059263000	2.266800000
1	-4.395494000	-6.970654000	2.502999000
6	-3.179078000	-7.554427000	-0.244195000
1	-3.555092000	-8.579734000	-0.274098000
1	-2.277433000	-7.493319000	-0.862105000
1	-2.896339000	-7.320871000	0.785476000
6	-4.496476000	-6.905253000	-2.280207000
1	-3.581052000	-6.707692000	-2.846703000
1	-4.782623000	-7.948868000	-2.415030000
1	-5.295797000	-6.275459000	-2.682003000
8	-6.090235000	-8.013622000	-0.310791000

Isotactic dimer S-PMTC-1^{mod} (UB3LYP/6-311+G**) Charge: 0, Multiplicity: 3

6	5.230947000	-0.110468000	-1.198130000
8	5.557236000	1.330669000	-1.201766000
6	5.501972000	2.180225000	-2.251169000
8	5.632884000	3.369584000	-2.069942000
16	5.257373000	1.474802000	-3.907475000
6	5.213580000	3.034476000	-4.870084000
1	6.148681000	3.584471000	-4.728680000
1	5.097333000	2.733551000	-5.917923000
1	4.364751000	3.647696000	-4.551929000
6	6.349011000	-0.924350000	-1.870083000
1	7.323910000	-0.571896000	-1.521017000
1	6.315231000	-0.864747000	-2.963986000
16	6.177689000	-2.722905000	-1.504246000
6	6.554589000	-2.876513000	0.261997000
8	6.280800000	-3.898927000	0.839056000
8	7.201719000	-1.785497000	0.731135000
6	7.300844000	-1.469111000	2.179164000
6	6.146973000	-0.519007000	2.499127000
1	6.193866000	-0.228238000	3.561773000
1	6.215243000	0.377574000	1.876435000
8	4.930935000	-1.236938000	2.231977000
6	7.429562000	-2.656693000	3.132100000
1	6.486174000	-3.197718000	3.233244000
1	8.204645000	-3.352550000	2.792279000
1	7.725681000	-2.250861000	4.111000000
6	3.691927000	-0.690373000	2.461211000
6	2.718414000	-1.582739000	2.921425000
6	3.315178000	0.641695000	2.120631000
6	1.382000000	-1.193429000	3.027314000
1	3.032187000	-2.599324000	3.149761000
6	1.981142000	1.028209000	2.257066000
6	0.999462000	0.114154000	2.688244000
1	0.628214000	-1.910488000	3.350755000
1	1.660718000	2.026798000	1.979578000
8	4.295113000	1.447548000	1.613288000
6	3.921835000	2.750173000	1.135561000
1	3.147712000	2.657380000	0.363974000
1	3.554315000	3.374459000	1.965161000
1	4.829360000	3.173973000	0.699541000
6	-0.423862000	0.464624000	2.732838000
1	-1.066363000	-0.370343000	3.055415000
7	-0.888832000	1.614924000	2.407107000
6	-2.337716000	1.856654000	2.437701000
6	-4.230495000	2.808429000	0.953854000
6	-4.740861000	0.999520000	2.813130000
7	-5.127366000	1.752204000	1.561198000
6	-4.603802000	2.943461000	-0.532681000
1	-5.650424000	3.243699000	-0.645111000
1	-3.956322000	3.706672000	-0.985831000
1	-4.438287000	1.990748000	-1.047047000
6	-4.473762000	4.155305000	1.670580000
1	-3.966144000	4.954213000	1.112248000
1	-5.549633000	4.368628000	1.696901000
1	-4.083627000	4.154300000	2.694367000
6	-5.534123000	-0.317503000	2.835411000
1	-5.233204000	-0.892532000	3.721612000
1	-6.610452000	-0.124963000	2.875168000
1	-5.315287000	-0.907450000	1.938247000
6	-5.119183000	1.840151000	4.054074000
1	-6.163121000	2.166052000	3.969091000
1	-5.012289000	1.219236000	4.954576000
1	-4.477237000	2.720264000	4.167787000
6	-2.759618000	2.362274000	1.048986000
1	-2.606159000	1.543299000	0.335846000
1	-2.125327000	3.208185000	0.750973000
6	-3.238418000	0.664560000	2.788574000
1	-2.984986000	0.251120000	3.775496000
1	-3.065937000	-0.124083000	2.041584000
1	-2.492462000	2.664272000	3.171348000
8	-6.381671000	1.835965000	1.304032000
6	3.793570000	-0.402391000	-1.672150000
1	3.321606000	0.501663000	-2.067660000
1	3.780142000	-1.186256000	-2.444722000
8	3.075206000	-0.880582000	-0.513469000
6	1.711537000	-0.995909000	-0.613547000
6	0.866300000	0.132447000	-0.812168000
6	1.140888000	-2.252154000	-0.391449000
6	-0.516382000	-0.045402000	-0.834241000
6	-0.248929000	-2.416093000	-0.381506000
1	1.813590000	-3.088257000	-0.212425000
6	-1.089896000	-1.314851000	-0.615826000
1	-1.183249000	0.791069000	-1.008107000
1	-0.682193000	-3.400716000	-0.206121000
8	1.481476000	1.347747000	-0.959130000
6	-2.549996000	-1.505578000	-0.657103000
7	-3.364364000	-0.537578000	-0.863072000
6	-4.805297000	-0.759753000	-0.997241000
6	-5.185870000	-0.406500000	-2.446990000
6	-5.350100000	-2.163053000	-0.704088000
6	-6.704017000	-0.388580000	-2.701424000
1	-4.710678000	-1.148066000	-3.107759000
6	-6.884870000	-2.263627000	-0.840524000
1	-4.889537000	-2.876072000	-1.405525000
7	-7.335529000	-1.646105000	-2.146799000
1	-2.879683000	-2.548808000	-0.512651000
1	-4.780736000	0.578175000	-2.714087000
1	-5.082591000	-2.487240000	0.312742000
1	-5.283406000	-0.033677000	-0.325211000
6	-6.954992000	-0.375261000	-4.220088000
1	-8.028856000	-0.334106000	-4.430011000
1	-6.465221000	0.508723000	-4.651044000
1	-6.536953000	-1.277749000	-4.685997000
6	-7.369223000	0.849099000	-2.058082000
1	-7.046249000	1.749049000	-2.600171000
1	-8.458874000	0.756089000	-2.144318000
1	-7.100114000	0.975132000	-1.004541000
6	-7.615336000	-1.560928000	0.325188000
1	-8.692824000	-1.564250000	0.119660000
1	-7.429674000	-2.116631000	1.254588000
1	-7.281424000	-0.529466000	0.472024000
6	-7.283352000	-3.750357000	-0.868970000
1	-6.932052000	-4.228152000	0.056115000
1	-8.370919000	-3.854301000	-0.940700000
1	-6.821952000	-4.254527000	-1.728840000
8	-8.545509000	-1.880916000	-2.498441000
1	5.260450000	-0.314197000	-0.126910000
1	8.223605000	-0.875785000	2.211013000
6	0.645445000	2.515488000	-0.878266000
1	-0.047243000	2.564083000	-1.732723000
1	0.080487000	2.510434000	0.065706000
1	1.331599000	3.368382000	-0.913333000

Atactic dimer PMTC-1^{mod} (UB97D/6-311+G**) Charge: 0, Multiplicity: 3

6	1.215444000	1.418351000	-0.836561000
8	1.119988000	0.136014000	-1.530595000
6	1.027345000	-1.075058000	-0.925453000
8	0.947370000	-2.072896000	-1.605375000
16	0.999472000	-1.113474000	0.885178000
6	0.548897000	-2.878696000	1.093944000
1	1.284612000	-3.510048000	0.586668000
1	0.567589000	-3.062275000	2.174869000
1	-0.451277000	-3.072179000	0.692333000
6	0.426103000	2.394309000	-1.724842000
1	-0.512799000	1.930235000	-2.036456000
1	1.003857000	2.658325000	-2.620908000
16	0.050415000	3.998538000	-0.896904000
6	-1.306660000	3.600296000	0.221433000
8	-1.787068000	4.448053000	0.936843000
8	-1.679642000	2.302903000	0.136417000
6	-2.823146000	1.866131000	0.965982000
1	-2.780957000	2.406241000	1.919128000
6	-2.671336000	0.366515000	1.158355000
1	-3.529682000	0.001519000	1.735017000
1	-2.661842000	-0.143598000	0.185802000
1	0.739464000	1.347025000	0.148055000
6	2.678719000	1.828183000	-0.705060000
1	3.205158000	1.575527000	-1.635036000
1	2.737708000	2.914204000	-0.530599000
8	3.262812000	1.153203000	0.425214000
6	4.585419000	0.784473000	0.316976000
6	4.991617000	-0.231166000	-0.588566000
6	5.521703000	1.359372000	1.184240000
6	6.337617000	-0.619525000	-0.613630000
6	6.859661000	0.959605000	1.164041000
1	5.170575000	2.122210000	1.877187000
6	7.278593000	-0.031070000	0.257995000
1	6.669182000	-1.394739000	-1.301782000
1	7.591887000	1.402977000	1.834266000
6	8.678979000	-0.486042000	0.183116000
1	8.862621000	-1.267164000	-0.575819000
7	9.600628000	-0.015565000	0.935864000
6	10.983432000	-0.478111000	0.813209000
6	11.287767000	-1.600635000	-0.188476000
6	11.858072000	0.730129000	0.436790000
6	12.760597000	-2.058071000	-0.159380000
1	11.047058000	-1.244347000	-1.202282000
6	13.369981000	0.439575000	0.497762000
1	11.584352000	1.038232000	-0.584129000
7	13.686473000	-0.862123000	-0.206740000
6	13.069119000	-2.887464000	1.108192000
1	12.782156000	-2.360753000	2.025228000
1	12.513813000	-3.834997000	1.065787000
1	14.143588000	-3.104307000	1.144217000
6	14.127758000	1.560713000	-0.235707000
1	13.824857000	1.598626000	-1.290584000
1	13.885246000	2.521235000	0.238991000
1	15.208354000	1.391276000	-0.183628000
6	13.045112000	-2.914187000	-1.406835000
1	14.079466000	-3.273323000	-1.398493000
1	12.360560000	-3.773621000	-1.410666000
1	12.882441000	-2.324406000	-2.318865000
6	13.867453000	0.356129000	1.958654000
1	14.916145000	0.034996000	1.966690000
1	13.792461000	1.348985000	2.423430000
1	13.274928000	-0.347323000	2.554870000
8	14.926860000	-1.112724000	-0.410867000
1	10.663188000	-2.485112000	0.005270000
1	11.269351000	-0.807970000	1.824046000
1	11.639552000	1.572760000	1.105731000
8	4.008189000	-0.762522000	-1.370946000
6	4.339326000	-1.851253000	-2.242015000
1	5.062523000	-1.532829000	-3.009825000
1	4.759576000	-2.692177000	-1.668046000
1	3.392838000	-2.147119000	-2.702930000
1	-1.742201000	0.134358000	1.691659000
6	-4.093373000	2.257122000	0.205926000
1	-4.108565000	3.340788000	0.017919000
1	-4.134992000	1.707838000	-0.744817000
8	-5.209848000	1.902354000	1.049437000
6	-6.437605000	1.920508000	0.424196000
6	-7.365639000	2.902606000	0.776402000
6	-6.796380000	0.921294000	-0.519148000
6	-8.650476000	2.912210000	0.216762000
1	-7.061796000	3.652403000	1.504832000
6	-8.080913000	0.941831000	-1.080317000
6	-9.021753000	1.915308000	-0.695540000
1	-9.366647000	3.679397000	0.507641000
1	-8.367957000	0.193629000	-1.811131000
8	-5.843244000	-0.018316000	-0.803160000
6	-6.209813000	-1.096453000	-1.674584000
1	-6.466959000	-0.723325000	-2.678420000
1	-5.328221000	-1.742195000	-1.731222000
1	-7.062175000	-1.658626000	-1.261864000
6	-10.405100000	1.912782000	-1.269363000
1	-10.646879000	2.817379000	-1.849387000
7	-11.350746000	1.055576000	-1.195921000
6	-11.314643000	-0.190103000	-0.414625000
6	-10.861335000	-1.369615000	-1.289236000
6	-10.513856000	-0.233847000	0.898074000
6	-11.120391000	-2.740694000	-0.631792000
1	-11.378697000	-1.347800000	-2.257945000
6	-10.786136000	-1.520315000	1.706390000
1	-9.440746000	-0.187358000	0.690250000
7	-10.652234000	-2.734292000	0.808942000
8	-10.609878000	-3.874218000	1.393768000
1	-9.784804000	-1.267497000	-1.480796000
1	-10.763626000	0.628410000	1.531493000
1	-12.371087000	-0.360827000	-0.164708000
6	-12.618584000	-3.115657000	-0.675437000
1	-12.920438000	-3.283170000	-1.718713000
1	-12.776552000	-4.038499000	-0.104275000
1	-13.251993000	-2.325367000	-0.257177000
6	-9.719270000	-1.642638000	2.809927000
1	-8.716348000	-1.700430000	2.366177000
1	-9.771200000	-0.752110000	3.450930000
1	-9.891645000	-2.538518000	3.415538000
6	-12.188467000	-1.519660000	2.354975000

1	-12.365793000	-2.491149000	2.832450000
1	-12.231000000	-0.732255000	3.120413000
1	-12.984800000	-1.333145000	1.625902000
6	-10.305739000	-3.812559000	-1.378592000
1	-10.601419000	-3.810652000	-2.436655000
1	-9.231498000	-3.592971000	-1.307680000
1	-10.490469000	-4.803104000	-0.950343000

Atactic dimer PMTC-1^{mod} (UB3LYP/6-311+G**) Charge: 0, Multiplicity: 3

6	-5.333254000	1.449558000	-1.290100000
1	-6.118685000	2.120467000	-1.657390000
8	-5.473138000	0.150202000	-1.927793000
6	-6.024456000	-0.035815000	-3.145527000
8	-6.080422000	-1.141764000	-3.626784000
16	-6.672943000	1.428061000	-3.977732000
6	-7.291396000	0.615171000	-5.490386000
1	-8.063319000	-0.113112000	-5.241687000
1	-7.710767000	1.414015000	-6.105453000
1	-6.472773000	0.124150000	-6.016617000
6	-5.544335000	1.151948000	0.197576000
1	-4.716770000	0.553775000	0.575714000
1	-6.478524000	0.601464000	0.321986000
16	-5.635187000	2.723233000	1.158468000
6	-5.172489000	2.228287000	2.816373000
8	-5.075969000	3.047649000	3.702503000
8	-4.973135000	0.907626000	2.932570000
6	-4.525179000	0.400043000	4.230050000
1	-3.910819000	1.175114000	4.695095000
6	-3.679160000	-0.821685000	3.923605000
1	-3.475170000	-1.361888000	4.858316000
1	-4.208919000	-1.485267000	3.231627000
8	-2.439478000	-0.385479000	3.347723000
6	-5.737467000	0.075801000	5.095111000
1	-5.413435000	-0.280873000	6.078209000
1	-6.344954000	0.971533000	5.243396000
1	-6.356883000	-0.698057000	4.629967000
6	-1.594060000	-1.385488000	2.917536000
6	-0.415147000	-1.620672000	3.617219000
6	-1.870246000	-2.111656000	1.730342000
6	0.500052000	-2.576015000	3.166080000
1	-0.224284000	-1.036627000	4.512193000
6	-0.957633000	-3.068272000	1.290020000
6	0.231374000	-3.306683000	2.003684000
1	1.419015000	-2.750576000	3.719112000
1	-1.130727000	-3.641999000	0.388351000
8	-3.033578000	-1.800358000	1.095486000
6	-3.298273000	-2.409682000	-0.172271000
1	-2.495704000	-2.189997000	-0.884113000
1	-3.418805000	-3.494400000	-0.068938000
1	-4.225149000	-1.964486000	-0.531103000
6	1.204985000	-4.314330000	1.542425000
1	2.098442000	-4.405276000	2.175097000
7	1.018729000	-5.021398000	0.496950000
6	1.997022000	-6.012528000	0.050620000
6	3.258405000	-6.746150000	-2.079539000
6	4.215477000	-7.283940000	0.343078000
7	4.355790000	-7.185684000	-1.150297000
6	3.927222000	-6.135614000	-3.322998000
1	4.523699000	-6.882141000	-3.850062000
1	3.155342000	-5.753248000	-3.998885000
1	4.583843000	-5.306466000	-3.041801000
6	2.425325000	-7.975928000	-2.504465000
1	1.725590000	-7.690520000	-3.296934000
1	3.088516000	-8.755296000	-2.888922000
1	1.842959000	-8.393574000	-1.678804000
6	5.617455000	-7.094124000	0.947767000
1	5.547131000	-7.108557000	2.040512000
1	6.290960000	-7.889554000	0.624840000
1	6.045915000	-6.135453000	0.639258000
6	3.691383000	-8.688908000	0.716929000
1	4.321588000	-9.450976000	0.251133000
1	3.730718000	-8.821037000	1.803487000
1	2.659150000	-8.851473000	0.396240000
6	2.384760000	-5.676806000	-1.396490000
1	2.920490000	-4.718595000	-1.388329000
1	1.479306000	-5.528610000	-1.993699000
6	3.283212000	-6.170428000	0.867787000
1	3.061624000	-6.398549000	1.917322000
1	3.830720000	-5.218506000	0.855055000
1	1.447858000	-6.964044000	0.050265000
8	5.280983000	-7.909343000	-1.674310000
6	-3.981476000	2.063992000	-1.640085000
1	-3.868010000	2.127132000	-2.725440000
1	-3.933135000	3.076792000	-1.221399000
8	-2.952401000	1.248254000	-1.071059000
6	-1.715888000	1.810422000	-0.867664000
6	-0.970395000	2.402317000	-1.910904000
6	-1.152686000	1.713121000	0.404857000
6	0.298004000	2.910413000	-1.654560000
6	0.127602000	2.208726000	0.652981000
1	-1.734029000	1.241690000	1.190656000
6	0.862480000	2.819619000	-0.371958000
1	0.879801000	3.372277000	-2.448550000
1	0.550607000	2.126886000	1.650299000
8	-1.541275000	2.438289000	-3.159740000
1	-0.927038000	2.851940000	-3.780618000
6	2.208625000	3.348037000	-0.088957000
7	2.923139000	3.920721000	-0.978111000
6	4.256814000	4.440551000	-0.677734000
6	4.280614000	5.930350000	-1.047863000
6	4.778387000	4.294589000	0.755305000
6	5.677989000	6.575427000	-0.978739000
1	3.600921000	6.457970000	-0.366070000
6	6.218374000	4.818956000	0.944253000
1	4.112031000	4.844016000	1.433580000
7	6.374579000	6.162313000	0.288014000
1	2.543106000	3.205444000	0.947355000
1	3.882027000	6.063548000	-2.058841000
1	4.768615000	3.246090000	1.076497000
1	4.925613000	3.902376000	-1.363369000
6	5.528690000	8.106000000	-0.942067000
1	6.505698000	8.592019000	-0.930840000
1	4.975171000	8.437660000	-1.826676000
1	4.977756000	8.420148000	-0.050197000
6	6.548359000	6.180025000	-2.192509000
1	6.125289000	6.612357000	-3.105374000
1	7.562107000	6.567321000	-2.060795000
1	6.607104000	5.097155000	-2.331308000
6	7.262159000	3.839922000	0.360821000
1	8.258598000	4.284467000	0.426779000
1	7.257569000	2.908383000	0.936855000
1	7.064652000	3.588909000	-0.684593000
6	6.492737000	5.009060000	2.446169000
1	6.338084000	4.058347000	2.967191000
1	7.517540000	5.343726000	2.614726000
1	5.813832000	5.753918000	2.872802000
8	7.454604000	6.801181000	0.570230000

PMTC-1^{mod} tetramer (UB3LYP/6-311+G**) Charge: 0, Multiplicity: 3

6	6.697549000	5.790956000	-0.178614000
1	6.889128000	6.785783000	0.240412000
8	6.729121000	5.866951000	-1.631051000
6	7.436663000	6.784416000	-2.321862000
8	7.441210000	6.769619000	-3.529248000
16	8.340192000	8.012970000	-1.355619000
6	9.043357000	8.968886000	-2.742538000
1	8.246925000	9.404916000	-3.345640000
1	9.643983000	9.757441000	-2.284514000
1	9.670680000	8.328666000	-3.362813000
6	5.263311000	5.353613000	0.134170000
1	5.100746000	4.339701000	-0.228704000
1	4.566677000	6.033780000	-0.358816000
16	4.971739000	5.392405000	1.954901000
6	3.574705000	4.294814000	2.176273000
8	3.194475000	3.982271000	3.278619000
8	3.032025000	3.905371000	1.005562000
6	1.965475000	2.909662000	1.039689000
1	2.030890000	2.361961000	1.982316000
6	2.223599000	1.987634000	-0.138674000
1	1.345053000	1.351516000	-0.301343000
1	2.419096000	2.578170000	-1.042035000
8	3.353769000	1.166856000	0.174873000
6	0.633301000	3.664875000	0.948017000
1	0.538990000	4.355765000	1.788006000
1	0.569886000	4.224320000	0.010186000
6	3.726003000	0.278873000	-0.813924000
6	3.445704000	-1.073880000	-0.654206000
6	4.452074000	0.729848000	-1.945629000
6	3.880414000	-1.998866000	-1.608585000
1	2.886404000	-1.391396000	0.220437000
6	4.876870000	-0.197401000	-2.895095000
6	4.595101000	-1.566666000	-2.731320000
1	3.660936000	-3.054664000	-1.474500000
1	5.437778000	0.104323000	-3.770604000
8	4.684018000	2.070480000	-2.010603000
6	5.511070000	2.564657000	-3.068409000
1	6.498914000	2.092513000	-3.042600000
1	5.042375000	2.394299000	-4.044887000
1	5.620513000	3.633335000	-2.889165000
6	5.045179000	-2.558769000	-3.725531000
1	4.778883000	-3.596534000	-3.482584000
7	5.688041000	-2.235224000	-4.779407000
6	6.132731000	-3.234933000	-5.749469000
6	8.246423000	-3.941045000	-7.054162000
6	6.278211000	-5.666691000	-6.579185000
7	7.678995000	-5.332913000	-7.010768000
6	9.767685000	-4.059336000	-6.859033000
1	10.221231000	-4.627243000	-7.673103000
1	10.208915000	-3.057603000	-6.833274000
1	9.999088000	-4.564474000	-5.916169000
6	7.959923000	-3.316138000	-8.437813000
1	8.498690000	-2.367488000	-8.532128000
1	8.302997000	-3.991959000	-9.225618000
1	6.896927000	-3.113783000	-8.594184000
6	6.298608000	-7.104660000	-6.032203000
1	5.302133000	-7.367053000	-5.661496000
1	6.585634000	-7.812747000	-6.811236000
1	7.011509000	-7.194935000	-5.206735000
6	5.337600000	-5.606461000	-7.804038000
1	5.744776000	-6.223252000	-8.609432000
1	4.350870000	-5.996138000	-7.531644000
1	5.204360000	-4.589328000	-8.181715000
6	7.652574000	-3.092330000	-5.913928000
1	8.122480000	-3.377390000	-4.963612000
1	7.905085000	-2.041670000	-6.089502000
6	5.818288000	-4.706406000	-5.460975000
1	4.741971000	-4.862264000	-5.319564000
1	6.309841000	-4.997283000	-4.523043000
1	5.653263000	-2.939944000	-6.692962000
8	8.251775000	-6.198488000	-7.770423000
6	7.786981000	4.845956000	0.320824000
1	8.762657000	5.170742000	-0.050372000
1	7.802855000	4.871965000	1.417219000
8	7.490395000	3.524164000	-0.136514000
6	7.983821000	2.461210000	0.583158000
6	9.372173000	2.281522000	0.805733000
6	7.081189000	1.500196000	1.029585000
6	9.815035000	1.159420000	1.500655000
6	7.533354000	0.369086000	1.712550000
1	6.024920000	1.652172000	0.833302000
6	8.899134000	0.194948000	1.960299000
1	10.866760000	0.990113000	1.694645000
1	6.817135000	-0.370416000	2.059606000
8	10.193517000	3.249558000	0.295149000
6	9.356204000	-0.996892000	2.696683000
7	10.586409000	-1.215551000	2.958862000
6	11.017502000	-2.402364000	3.696843000
6	11.817599000	-1.934422000	4.920755000
6	9.940700000	-3.381638000	4.175458000
6	12.513157000	-3.071186000	5.693384000
1	11.128023000	-1.405652000	5.591631000
6	10.510740000	-4.626824000	4.888774000
1	9.266616000	-2.857939000	4.866278000
7	11.565659000	-4.223390000	5.880914000
1	8.550249000	-1.679098000	2.998632000
1	12.574675000	-1.208032000	4.607844000
1	9.327538000	-3.737451000	3.338854000
1	11.702389000	-2.922589000	3.013095000
6	12.905549000	-2.567347000	7.092891000
1	13.432894000	-3.342383000	7.651631000
1	13.556765000	-1.692646000	6.994049000
1	12.017645000	-2.276041000	7.662490000
6	13.779484000	-3.566516000	4.959536000
1	14.536320000	-2.775034000	4.955928000

1	14.188560000	-4.437890000	5.477643000
1	13.580293000	-3.843614000	3.920826000
6	11.113715000	-5.633289000	3.882884000
1	11.588768000	-6.454336000	4.426125000
1	10.319211000	-6.046290000	3.252217000
1	11.859229000	-5.175992000	3.227083000
6	9.383078000	-5.317361000	5.675465000
1	8.571594000	-5.583432000	4.990020000
1	9.747607000	-6.222939000	6.163079000
1	8.982873000	-4.650083000	6.445077000
8	11.951956000	-5.142954000	6.693100000
6	11.599649000	3.099048000	0.467554000
1	11.872805000	3.095036000	1.529647000
1	11.960552000	2.178325000	-0.006001000
1	12.051498000	3.962285000	-0.022547000
16	-0.834291000	2.565162000	0.986731000
6	-1.207432000	2.645016000	2.762656000
8	-0.683285000	3.431244000	3.509065000
8	-2.134284000	1.783210000	3.223978000
6	-2.793695000	0.761464000	2.440306000
1	-2.419483000	0.760593000	1.414909000
6	-2.515244000	-0.586572000	3.106199000
1	-2.749228000	-0.524500000	4.173526000
1	-3.133724000	-1.365815000	2.658008000
6	-4.284843000	1.069910000	2.423897000
1	-4.637911000	1.263005000	3.445330000
1	-4.836958000	0.223449000	2.004156000
8	-4.468081000	2.235026000	1.612846000
6	-5.679841000	2.882339000	1.692716000
6	-5.695341000	4.204628000	2.127981000
6	-6.884671000	2.265873000	1.284637000
6	-6.889371000	4.925914000	2.170516000
1	-4.756438000	4.651018000	2.439231000
6	-8.075311000	2.998393000	1.314789000
6	-8.087413000	4.338007000	1.745280000
1	-6.887578000	5.951132000	2.529452000
1	-9.005125000	2.527376000	1.022965000
8	-6.787744000	0.964831000	0.877692000
6	-7.978644000	0.272224000	0.507763000
1	-8.451018000	0.734018000	-0.366724000
1	-7.667465000	-0.743314000	0.261969000
1	-8.693594000	0.241641000	1.338750000
6	-9.336857000	5.150426000	1.835322000
1	-9.416172000	5.725279000	2.765104000
7	-10.300648000	5.315584000	1.013854000

6	-10.323927000	4.721694000	-0.320899000
6	-11.764488000	3.212397000	-1.845538000
6	-10.716544000	5.382250000	-2.785702000
7	-11.822337000	4.379386000	-2.772294000
8	-11.979791000	3.867935000	-4.119397000
1	-9.393974000	4.188666000	-0.554881000
6	-9.386376000	4.885325000	-3.401878000
1	-8.728056000	5.741822000	-3.583441000
1	-8.844966000	4.192441000	-2.755165000
1	-9.573234000	4.392070000	-4.359097000
6	-11.214453000	6.577881000	-3.622762000
1	-10.513296000	7.414798000	-3.537165000
1	-11.293215000	6.311617000	-4.680618000
1	-12.195408000	6.909619000	-3.267955000
6	-10.718450000	2.134359000	-2.220477000
1	-9.691343000	2.451562000	-2.030098000
1	-10.903801000	1.228298000	-1.632309000
1	-10.806783000	1.874375000	-3.278214000
6	-13.158846000	2.554049000	-1.870870000
1	-13.350337000	2.075642000	-2.835394000
1	-13.227958000	1.785328000	-1.093689000
1	-13.936220000	3.302552000	-1.688251000
6	-10.525495000	5.861781000	-1.330924000
1	-9.669431000	6.545504000	-1.298202000
1	-11.406379000	6.433202000	-1.016672000
6	-11.522831000	3.768278000	-0.424296000
1	-12.412060000	4.317984000	-0.094794000
1	-11.401195000	2.921703000	0.263034000
16	-0.761546000	-1.129932000	3.056327000
6	-0.557764000	-1.780715000	1.403116000
8	-1.654449000	-1.658094000	0.635626000
8	0.494091000	-2.275745000	1.060341000
6	-1.595981000	-2.225087000	-0.716534000
1	-0.995008000	-3.136337000	-0.663691000
6	-0.973798000	-1.218554000	-1.676802000
1	0.045908000	-0.979110000	-1.368672000
1	-0.929139000	-1.643604000	-2.684763000
1	-1.560841000	-0.295088000	-1.714478000
6	-3.027829000	-2.569560000	-1.084089000
1	-3.680181000	-1.704881000	-0.917771000
1	-3.076932000	-2.844141000	-2.141676000
8	-3.446883000	-3.676180000	-0.272039000
6	-4.757640000	-3.773439000	0.108923000
6	-5.017654000	-4.071258000	1.445483000
6	-5.838364000	-3.674854000	-0.806157000

6	-6.326699000	-4.250412000	1.896921000
1	-4.171131000	-4.170542000	2.117284000
6	-7.143497000	-3.829170000	-0.344979000
6	-7.400014000	-4.117230000	1.009201000
1	-6.510752000	-4.487001000	2.941333000
1	-7.991116000	-3.761069000	-1.015741000
8	-5.502784000	-3.451652000	-2.111974000
6	-6.547681000	-3.403362000	-3.081418000
1	-6.052896000	-3.245993000	-4.040261000
1	-7.106723000	-4.345861000	-3.106936000
1	-7.236645000	-2.573473000	-2.884536000
6	-8.776699000	-4.300324000	1.496782000
1	-8.864008000	-4.589971000	2.557673000
7	-9.809357000	-4.144178000	0.763086000
6	-11.122212000	-4.374095000	1.354611000
6	-11.962774000	-3.094872000	1.247099000
6	-11.834970000	-5.491956000	0.581259000
6	-13.414777000	-3.256425000	1.749238000
1	-11.486025000	-2.282040000	1.807642000
6	-13.281361000	-5.755977000	1.054289000
1	-11.852259000	-5.207095000	-0.476908000
7	-13.987903000	-4.444934000	1.059269000
8	-15.359211000	-4.616924000	1.416912000
1	-11.981544000	-2.795162000	0.192966000
1	-11.263927000	-6.424612000	0.655503000
1	-11.016474000	-4.662477000	2.414375000
6	-14.227241000	-2.033107000	1.279507000
1	-13.764117000	-1.110978000	1.647718000
1	-14.260886000	-2.001375000	0.186944000
1	-15.254402000	-2.075838000	1.648910000
6	-13.458773000	-3.299128000	3.299391000
1	-12.791413000	-4.046657000	3.732343000
1	-13.161600000	-2.323094000	3.696926000
1	-14.472626000	-3.489125000	3.669030000
6	-13.981243000	-6.636088000	-0.000564000
1	-14.042587000	-6.103254000	-0.953516000
1	-13.417640000	-7.563696000	-0.148904000
1	-14.996575000	-6.892898000	0.310252000
6	-13.286705000	-6.521618000	2.403502000
1	-14.303562000	-6.649052000	2.791597000
1	-12.885050000	-7.528430000	2.249232000
1	-12.683013000	-6.042888000	3.176895000
1	-12.854413000	4.190025000	-4.375117000
1	-15.392776000	-4.884072000	2.353762000

PMTC-1^{mod} tetramer (UB97D/6-311+G**) Charge: 0, Multiplicity: 3

6	6.574239000	5.547921000	1.151784000
1	7.200093000	6.291578000	1.671987000
8	7.293878000	5.041501000	-0.018451000
6	8.602128000	4.709130000	-0.018451000
8	9.098485000	4.187398000	-0.999177000
16	9.566717000	5.122251000	1.466060000
6	11.188605000	4.483891000	0.893094000
1	11.473013000	4.976661000	-0.043134000
1	11.906258000	4.720842000	1.688742000
1	11.131066000	3.400069000	0.739979000
6	5.346403000	6.220094000	0.512510000
1	4.751744000	5.451027000	0.010579000
1	5.686428000	6.960742000	-0.221795000
16	4.289210000	7.045394000	1.787056000
6	2.604025000	6.648564000	1.272136000
8	1.669118000	6.764447000	2.034745000
8	2.547155000	6.263885000	-0.028952000
6	1.356230000	5.519773000	-0.454767000
1	0.801138000	5.202287000	0.437713000
6	1.897917000	4.311532000	-1.207907000
1	1.072566000	3.775996000	-1.706303000
1	2.632425000	4.633570000	-1.959027000
8	2.502649000	3.464071000	-0.215579000
6	0.474711000	6.432881000	-1.315231000
1	0.218189000	7.345633000	-0.763363000
1	0.981071000	6.703118000	-2.252144000
6	3.196610000	2.359264000	-0.659316000
6	2.772098000	1.113374000	-0.188434000
6	4.375687000	2.453973000	-1.457383000
6	3.486698000	-0.043497000	-0.495232000
1	1.900036000	1.080277000	0.460873000
6	5.081995000	1.285281000	-1.772697000
6	4.646043000	0.035608000	-1.282597000
1	3.176246000	-1.012981000	-0.117344000
1	5.992927000	1.317575000	-2.364495000

8	4.742483000	3.711054000	-1.850124000
6	5.950301000	3.834095000	-2.620255000
1	6.817394000	3.476147000	-2.050371000
1	5.860346000	3.281393000	-3.570454000
1	6.063605000	4.905623000	-2.815622000
6	5.367737000	-1.219311000	-1.514595000
1	4.824501000	-2.101278000	-1.140315000
7	6.525860000	-1.289359000	-2.070123000
6	7.156401000	-2.609908000	-2.196923000
6	8.917013000	-4.139288000	-1.107807000
6	6.970696000	-5.142307000	-2.598796000
7	8.064842000	-5.304931000	-1.567046000
6	9.497579000	-4.509117000	0.268643000
1	10.136498000	-5.396277000	0.189423000
1	10.087866000	-3.662165000	0.646509000
1	8.685718000	-4.718873000	0.975250000
6	10.081854000	-3.938418000	-2.105560000
1	10.818874000	-3.247674000	-1.669742000
1	10.565007000	-4.906589000	-2.292304000
1	9.743641000	-3.516454000	-3.059799000
6	5.993114000	-6.321961000	-2.442399000
1	5.203786000	-6.233466000	-3.201924000
1	6.519730000	-7.274998000	-2.565369000
1	5.523509000	-6.306196000	-1.449735000
6	7.587518000	-5.199059000	-4.016064000
1	8.214063000	-6.097446000	-4.093943000
1	6.782185000	-5.256999000	-4.763166000
1	8.200046000	-4.318371000	-4.241404000
6	8.057171000	-2.864166000	-0.972402000
1	7.406898000	-2.936376000	-0.084839000
1	8.735155000	-2.011116000	-0.818797000
6	6.217732000	-3.823534000	-2.338536000
1	5.495031000	-3.661612000	-3.152482000
1	5.643919000	-3.949797000	-1.407377000
1	7.790645000	-2.551993000	-3.095005000

8	8.608166000	-6.469005000	-1.490327000
6	6.254304000	4.414407000	2.131522000
1	7.163894000	3.828935000	2.318913000
1	5.910901000	4.861246000	3.079931000
8	5.207766000	3.595658000	1.590272000
6	5.226635000	2.260605000	1.924544000
6	6.219678000	1.386724000	1.390773000
6	4.183126000	1.747878000	2.699994000
6	6.109084000	0.012162000	1.618053000
6	4.076073000	0.368263000	2.918544000
1	3.438436000	2.447730000	3.077242000
6	5.025426000	-0.507564000	2.360561000
1	6.806024000	-0.691008000	1.170793000
1	3.238693000	-0.034506000	3.491051000
8	7.198611000	1.977892000	0.645719000
6	4.800623000	-1.955677000	2.463610000
7	5.548071000	-2.824503000	1.879009000
6	5.227350000	-4.248909000	1.967556000
6	6.292512000	-4.967467000	2.815315000
6	3.857857000	-4.653343000	2.537867000
6	6.196343000	-6.505468000	2.725346000
1	6.159945000	-4.646930000	3.861656000
6	3.580351000	-6.163747000	2.403724000
1	3.823969000	-4.384405000	3.606019000
7	4.761755000	-6.955912000	2.912435000
1	3.903892000	-2.231983000	3.046320000
1	7.300354000	-4.660686000	2.506511000
1	3.048127000	-4.105414000	2.038451000
1	5.312483000	-4.623317000	0.933208000
6	7.023915000	-7.133757000	3.861558000
1	6.959379000	-8.226891000	3.814896000
1	8.073058000	-6.823221000	3.753752000
1	6.650276000	-6.796615000	4.839219000
6	6.707622000	-7.026096000	1.364480000
1	7.790876000	-6.872623000	1.276427000

1	6.488762000	-8.098807000	1.286683000
1	6.235958000	-6.507403000	0.523157000
6	3.292219000	-6.569734000	0.937449000
1	3.256600000	-7.664966000	0.873156000
1	2.317008000	-6.169378000	0.617202000
1	4.072347000	-6.214795000	0.249282000
6	2.364710000	-6.535956000	3.273615000
1	1.490831000	-5.963482000	2.931493000
1	2.157213000	-7.609294000	3.194937000
1	2.563085000	-6.290389000	4.326615000
8	4.577362000	-8.223305000	3.033807000
6	8.161032000	1.112082000	0.015506000
1	8.716406000	0.534054000	0.774376000
1	7.667174000	0.422078000	-0.685529000
1	8.837411000	1.784318000	-0.521690000
16	-1.105068000	5.577939000	-1.738884000
6	-2.259926000	6.531996000	-0.699600000
8	-2.018088000	7.625083000	-0.239589000
8	-3.497235000	5.981648000	-0.582705000
6	-3.757104000	4.552325000	-0.363055000
1	-2.811069000	4.005629000	-0.305265000
6	-4.531271000	4.467560000	0.970811000
1	-5.023421000	5.427916000	1.172894000
1	-5.288468000	3.675995000	0.937784000
6	-4.570098000	3.989808000	-1.541884000
1	-5.212043000	4.782102000	-1.954408000
1	-5.197341000	3.156630000	-1.193503000
8	-3.711935000	3.537326000	-2.618284000
6	-3.552113000	2.169040000	-2.694684000
6	-4.454998000	1.410215000	-3.446557000
6	-2.476444000	1.511596000	-2.032566000
6	-4.303828000	0.023994000	-3.565012000
1	-5.275488000	1.931785000	-3.939072000
6	-2.330920000	0.117268000	-2.153379000
6	-3.234898000	-0.651092000	-2.934025000
1	-5.019483000	-0.547239000	-4.157666000
1	-1.538853000	-0.367128000	-1.595737000
8	-1.656861000	2.309796000	-1.296284000
6	-0.559059000	1.710147000	-0.601056000
1	0.120110000	1.198105000	-1.300258000
1	-0.026216000	2.532589000	-0.113944000
1	-0.914022000	0.992165000	0.153089000
6	-3.203111000	-2.125661000	-3.152300000
1	-4.137709000	-2.474700000	-3.621105000
7	-2.346004000	-3.063404000	-2.952174000
6	-1.041150000	-2.832052000	-2.347729000
6	0.218630000	-3.040952000	-0.103169000
6	1.402536000	-3.554360000	-2.360333000
7	1.276954000	-3.757975000	-0.877627000
8	2.563990000	-3.417492000	-0.270855000
1	-0.688051000	-1.797943000	-2.468059000
6	1.997836000	-2.197536000	-2.808041000
1	2.180962000	-2.231599000	-3.892372000
1	1.337050000	-1.345447000	-2.612359000
1	2.948723000	-2.013051000	-2.302105000
6	2.340254000	-4.675196000	-2.860755000
1	2.392620000	-4.656219000	-3.959243000
1	3.354900000	-4.531720000	-2.466185000
1	1.964252000	-5.656326000	-2.537349000
6	0.491470000	-1.545305000	0.178484000
1	0.634668000	-0.961851000	-0.735354000
1	-0.364058000	-1.130412000	0.730301000
1	1.382833000	-1.432081000	0.804869000
6	0.083504000	-3.758976000	1.257532000
1	0.991311000	-3.624491000	1.859504000
1	-0.765046000	-3.338726000	1.818368000
1	-0.088797000	-4.833085000	1.101532000
6	0.002464000	-3.764622000	-2.981581000
1	0.073971000	-3.586079000	-4.064244000
1	-0.324115000	-4.805202000	-2.832710000
6	-1.124030000	-3.201437000	-0.855378000
1	-1.458248000	-4.248619000	-0.798141000
1	-1.878183000	-2.586549000	-0.343830000
16	-3.469597000	4.135306000	2.449722000
6	-3.406155000	2.333917000	2.469422000
8	-3.786196000	1.794663000	1.279410000
8	-3.066093000	1.715325000	3.458284000
6	-4.326120000	0.423827000	1.326245000
1	-4.946749000	0.344548000	2.226837000
6	-3.205252000	-0.613811000	1.336310000
1	-2.565840000	-0.458933000	2.212755000
1	-3.637902000	-1.624545000	1.382193000
1	-2.607432000	-0.534463000	0.419812000
6	-5.198508000	0.303177000	0.084454000
1	-4.599398000	0.464111000	-0.819679000
1	-5.631794000	-0.704985000	0.054226000
8	-6.237215000	1.315330000	0.096032000
6	-7.540587000	0.893027000	-0.037912000
6	-8.307671000	1.435534000	-1.074628000
6	-8.141426000	-0.008275000	0.891587000
6	-9.661176000	1.098820000	-1.212136000
1	-7.819530000	2.122976000	-1.765060000
6	-9.487000000	-0.353081000	0.738740000
6	-10.257157000	0.197426000	-0.312202000
1	-10.253851000	1.525781000	-2.022643000
1	-9.984593000	-1.034953000	1.424531000
8	-7.326652000	-0.468769000	1.894814000
6	-7.910386000	-1.355414000	2.857656000
1	-7.109519000	-1.590161000	3.568082000
1	-8.749066000	-0.869443000	3.382421000
1	-8.273110000	-2.279046000	2.376861000
6	-11.676597000	-0.158071000	-0.469408000
1	-12.194884000	0.334563000	-1.318393000
7	-12.280757000	-0.976133000	0.314061000
6	-13.690566000	-1.247990000	0.063014000
6	-13.880080000	-2.756972000	-0.173228000
6	-14.508862000	-0.855825000	1.306220000
6	-15.360397000	-3.161558000	-0.364610000
1	-13.309180000	-3.080640000	-1.056514000
6	-16.013747000	-1.185914000	1.173031000
1	-14.096377000	-1.405286000	2.165734000
7	-16.121962000	-2.630827000	0.806628000
8	-17.501852000	-3.016944000	0.716226000
1	-13.474379000	-3.286564000	0.701854000
1	-14.400644000	0.219786000	1.511513000
1	-14.062966000	-0.685467000	-0.817636000
6	-15.454119000	-4.701313000	-0.294564000
1	-14.812997000	-5.147773000	-1.069325000
1	-15.123388000	-5.047273000	0.694096000
1	-16.488997000	-5.030529000	-0.450711000
6	-15.868947000	-2.707132000	-1.760065000
1	-15.667869000	-1.650050000	-1.966573000
1	-15.365205000	-3.302054000	-2.535670000
1	-16.948912000	-2.889554000	-1.869359000
6	-16.668152000	-1.030022000	2.562961000
1	-16.216300000	-1.742462000	3.266356000
1	-16.513220000	-0.005585000	2.933195000
1	-17.744944000	-1.232427000	2.505405000
6	-16.701299000	-0.191279000	0.198185000
1	-17.746340000	-0.479086000	0.006606000
1	-16.725255000	0.805524000	0.661868000
1	-16.179572000	-0.101088000	-0.761031000
1	2.930002000	-4.293691000	-0.075416000
1	-17.874542000	-2.539538000	-0.053687000

Isotactic dimer of GTEMPO-PMTCs (UB97D/6-311+G**) Charge: 0, Multiplicity: 3

6	0.371195000	2.778404000	0.401576000
1	0.249804000	3.871160000	0.457227000
8	-0.032386000	2.181136000	1.678449000
6	-1.136782000	2.548129000	2.370254000
8	-1.481203000	1.925756000	3.345698000
16	-2.030117000	4.018756000	1.777070000
6	-3.350211000	4.051636000	3.048991000
1	-2.911446000	4.197243000	4.040593000
1	-3.995997000	4.897467000	2.785770000
1	-3.914711000	3.114432000	3.023041000
6	1.858650000	2.413307000	0.285160000
1	1.966285000	1.348275000	0.065226000
1	2.369975000	2.655675000	1.222296000
16	2.621495000	3.380432000	-1.101584000
6	3.952430000	2.313726000	-1.690253000
8	4.492541000	2.519682000	-2.753018000
8	4.239174000	1.336552000	-0.811784000
6	5.082456000	0.220955000	-1.285414000
1	4.852895000	0.060303000	-2.345719000
6	4.628530000	-0.975746000	-0.461447000
1	5.272332000	-1.844067000	-0.699609000
1	4.753759000	-0.736896000	0.607987000
8	3.271524000	-1.246974000	-0.780379000
6	6.556140000	0.558646000	-1.086646000
1	7.176761000	-0.281392000	-1.428795000
1	6.817969000	1.447619000	-1.670778000
1	6.769198000	0.745964000	-0.025203000
6	1.231640000	-2.417032000	-0.486547000
6	2.544273000	-1.702930000	1.543734000
6	0.502095000	-3.569640000	0.235202000
1	0.681822000	-1.474035000	-0.398135000
6	1.343544000	-2.275607000	2.361854000
6	-0.493992000	2.235398000	-0.741152000
1	-1.533770000	2.546027000	-0.578247000
1	-0.145481000	2.693490000	-1.681360000
8	-0.365456000	0.817774000	-0.802731000
6	-1.575506000	0.082304000	-1.071244000
6	-2.556858000	0.126397000	0.110154000
6	-2.278520000	0.491086000	-2.366198000
6	-3.983021000	-0.408815000	-0.233517000
6	-3.294827000	-0.598962000	-2.777225000
1	-2.786728000	1.456125000	-2.231168000
1	2.453753000	-0.607930000	1.517611000
1	3.480210000	-1.942482000	2.065175000
1	1.300634000	-2.657031000	-1.555675000
1	-1.215417000	-0.944578000	-1.187526000
1	-2.132128000	-0.443714000	0.942902000
1	-2.659086000	1.161516000	0.460051000
1	-1.545079000	0.610791000	-3.174485000
6	-4.996602000	0.751546000	-0.375521000
1	-5.115707000	1.250837000	0.596872000
1	-4.655137000	1.497662000	-1.103317000
1	-5.968813000	0.355628000	-0.695682000
6	-4.453217000	-1.365200000	0.872928000
1	-3.791156000	-2.238054000	0.929439000
1	-4.413201000	-0.827816000	1.830300000
1	-5.475440000	-1.709873000	0.687368000
6	-4.375420000	-0.017211000	-3.709660000
1	-3.899695000	0.356651000	-4.627098000
1	-5.097881000	-0.799304000	-3.970675000
1	-4.905758000	0.811903000	-3.225307000
6	-2.574132000	-1.764460000	-3.485110000
1	-2.162661000	-1.409036000	-4.439308000
1	-1.752466000	-2.154735000	-2.870799000
1	-3.286473000	-2.574768000	-3.675442000
6	-1.024605000	-3.439853000	0.065371000
1	-1.380330000	-2.484616000	0.465509000
1	-1.285927000	-3.494621000	-1.000046000
1	-1.526266000	-4.256320000	0.597506000
6	0.970270000	-4.933159000	-0.310879000
1	0.664635000	-5.026300000	-1.361858000
1	2.062716000	-5.027710000	-0.250137000
1	0.513598000	-5.740339000	0.272559000
6	0.182379000	-1.257268000	2.436355000
1	0.480384000	-0.395596000	3.046686000
1	-0.086800000	-0.875509000	1.447836000
1	-0.692616000	-1.730352000	2.900427000
6	1.804510000	-2.620922000	3.785570000
1	0.963230000	-2.961097000	4.397803000
1	2.565256000	-3.412187000	3.762926000
1	2.239098000	-1.716785000	4.233766000
6	2.628015000	-2.200121000	0.082195000
1	3.194089000	-3.148336000	0.050037000
8	-4.843410000	-2.071299000	-1.733007000
8	0.277373000	-4.425598000	2.443459000
7	0.839903000	-3.541212000	1.707922000
7	-3.953349000	-1.172239000	-1.539778000
